# Evolutionary Genomics and Adaptive Evolution of the Hedgehog Gene Family (*Shh, Ihh and Dhh*) in Vertebrates

**DOI:** 10.1371/journal.pone.0074132

**Published:** 2014-12-30

**Authors:** Joana Pereira, Warren E. Johnson, Stephen J. O’Brien, Erich D. Jarvis, Guojie Zhang, M. Thomas P. Gilbert, Vitor Vasconcelos, Agostinho Antunes

**Affiliations:** 1 CIIMAR/CIMAR, Interdisciplinary Centre of Marine and Environmental Research, University of Porto, Porto, Portugal; 2 Smithsonian Conservation Biology Institute, National Zoological Park, Front Royal, Virginia, United States of America; 3 Theodosius Dobzhansky Center for Genome Bioinformatics, St. Petersburg State University, St. Petersburg, Russia; 4 Oceanographic Center, N. Ocean Drive, Nova Southeastern University, Ft. Lauderdale, Florida, United States of America; 5 Howard Hughes Medical Institute, Department of Neurobiology, Duke University Medical Center, Durham, North Carolina, United States of America; 6 BGI-Shenzhen, Beishan Industrial Zoon, Yantian District, Shenzhen, China; 7 Centre for GeoGenetics, Natural History Museum of Denmark, University of Copenhagen, Copenhagen, Denmark; 8 Department of Biology, Faculty of Sciences, University of Porto, Porto, Portugal; Laboratoire Arago, France

## Abstract

The Hedgehog (*Hh*) gene family codes for a class of secreted proteins composed of two active domains that act as signalling molecules during embryo development, namely for the development of the nervous and skeletal systems and the formation of the testis cord. While only one *Hh* gene is found typically in invertebrate genomes, most vertebrates species have three (Sonic hedgehog – *Shh*; Indian hedgehog – *Ihh*; and Desert hedgehog – *Dhh*), each with different expression patterns and functions, which likely helped promote the increasing complexity of vertebrates and their successful diversification. In this study, we used comparative genomic and adaptive evolutionary analyses to characterize the evolution of the *Hh* genes in vertebrates following the two major whole genome duplication (WGD) events. To overcome the lack of *Hh*-coding sequences on avian publicly available databases, we used an extensive dataset of 45 avian and three non-avian reptilian genomes to show that birds have all three *Hh* paralogs. We find suggestions that following the WGD events, vertebrate *Hh* paralogous genes evolved independently within similar linkage groups and under different evolutionary rates, especially within the catalytic domain. The structural regions around the ion-binding site were identified to be under positive selection in the signaling domain. These findings contrast with those observed in invertebrates, where different lineages that experienced gene duplication retained similar selective constraints in the *Hh* orthologs. Our results provide new insights on the evolutionary history of the *Hh* gene family, the functional roles of these paralogs in vertebrate species, and on the location of mutational hotspots.

## Introduction

**Figure 1:**
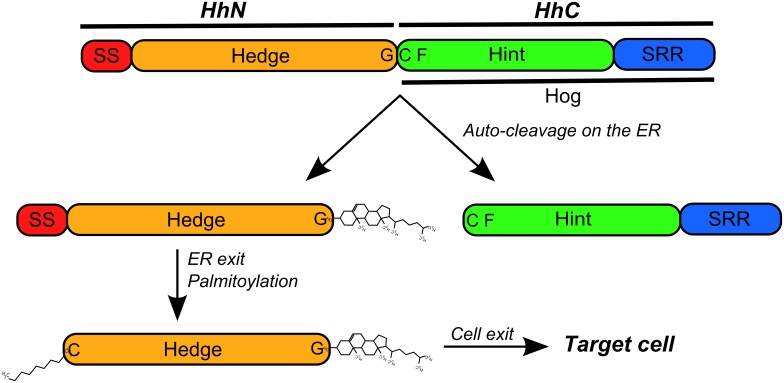
Structural features of the Hh proteins. The hedgehog proteins are composed by two main domains: the Hedge (N-terminal) and Hog (C-terminal) domains. The Hedge domain forms the HhN portion of the Hh proteins (together with the signaling sequence, SS) that is separated from the rest of protein by an auto-cleavage reaction preformed by the Hog domain [4]. The Hog domain forms the HhC portion of the Hh proteins and shares similarity with self-splicing Inteins on the Hint module [22]. The auto-cleavage reaction occurs on a GCF (glycine-cysteine-phenylalanine) motif that forms the boundary between the two main parts of the Hh proteins, with the cysteine residue initiating a nucleophilic attack on the carbonyl carbon of the preceding residue, the glycine [5]. The sterol-recognition region (SRR) forms the C-terminal region of the Hog domain and binds a cholesterol moiety that acts as an electron donor on a second nucleophilic attack that results in the cleavage of the bound between the glycine and cystein residues and in the attachment of the cholesterol moiety to the glycine residue [5]. After auto-cleavage, the sterified HhN fragment is further palmitoylated on the cystein residue immediately after the SS region [13] and leaves the endoplasmic reticulum (ER) for later export [11].

Cell-to-cell signaling is a process crucial to the development and survival of multicellular organisms, and is controlled by only a few signaling pathways that interact with molecules that are responsible for many of the diverse and complex functions observed in modern vertebrates [1]. Metazoans use numerous signaling proteins for cell-to-cell communication, all of which are encoded by a small number of gene families, among which the Hedgehog (Hh) signaling pathway is “one of the most enigmatic” [1], [2]. The Hedgehog (*Hh*) gene family codes for a class of secreted proteins that act as key modulators during embryogenesis and homeostasis of adult tissues in vertebrates and invertebrates [3]. Hh proteins are synthesized as pro-proteins that undergo auto-cleavage [4] and lipid modifications [5], [6] in the endoplasmic reticulum (ER) to produce a mature signaling peptide [7] that leaves the cell and can be incorporated into lipoprotein particles [8] or diffuse freely to target cells [2]. This is possible because Hh proteins are composed of two distinct domains: a N-terminal “Hedge” domain and a C-terminal “Hog” domain [9], separated during an auto-cleavage reaction to generate two similar-sized globular fragments [4], [9], HhN and HhC (Fig. 1).

The Hog domain is highly conserved [10] and promotes the auto-cleavage reaction and the addition of a cholesterol moiety to the signaling peptide HhN on the endoplasmic reticulum (ER) [9], [11] (Fig. 1). The Hog reaction is similar to the protein-splicing activity of Inteins and requires the “Hint” region and a sterol recognition region. The Hint region forms the N-terminal part of the Hog domain [12] and encompasses a highly conserved glycine-cysteine-phenylalanine (GCF) motif, and the sterol recognition region (SRR) on the Hog domain C-terminal end recruits cholesterol that acts as an electron donor on the auto-cleavage reaction and becomes incorporated into the signaling HhN peptide [5]. The Hedge domain is also highly conserved [10] and, together with a signaling sequence (SS), forms the HhN peptide after cleavage [9]. Before leaving the ER and separating from the SS, the HhN peptide undergoes further palmitoylation at its N-terminal [6], [13] and leaves the cell to act as a long- and short-range signal molecule recognized by transmembrane co-receptors, including the Interference hedgehog (IHog), Brother of interference hedgehog (BOI) and their homologs [14]. These co-receptors present the signaling HhN peptide to the transmembrane receptor Patched (Ptc), subsequently activating the Ci/Gli transcription factors [15]. This signaling activity has important roles in differentiation, survival and cell cycle progression [3], [16], which links the Hedgehog signaling pathway to several congenital and hereditary diseases, including holoprosencephaly and cyclopia [17], [18], acrocapitofemoral dysplasia [19], and gonadal dysgenesis with minifascicular neuropathy [20], and links it to tumerogenesis, including basal cell carcinoma, medulloblastoma, breast and liver cancers [16], [21].

Hog and Hedge-related proteins, as well Hh pathway proteins, are found in many organisms from a wide range of phyla [2], but proteins with both the Hedge and Hog domains are found only in the eumetazoa [22]. Most bilaterians, with the exception of *C. elegans* [22], [23], have been shown to possess at least one *Hh* gene, with the genome expansions in vertebrates giving rise to at least three *Hh*: *Shh, Ihh* and *Dhh.* In *Drosophila,* the only *Hh* gene is expressed in different developing embryo stages and tissues [24]. In contrast, in the vertebrates, each *Hh* gene has different roles which are reflected in their expression patterns [25]: *Shh* has a central role in the development and patterning of the nervous and skeletal systems [3], *Ihh* mediates endochondral bone formation and vasculangiogenesis, and *Dhh* is essential for the formation of the peripheral neurvous system [26] and is involved in the differentiation of peritubular myoid cells and consequent formation of the testis cord [27].

Two Whole-Genome Duplications (WGD) that occurred prior to the emergence of chordates seem to have led to the emergence of the *Hh* vertebrate paralogous genes. The current hypothesis is that the first duplication around 660 million years ago (mya) of an ancestral *Hh* gene gave rise to the ancestral *Shh*/*Ihh* and *Dhh* genes and an additional duplication event around 560 mya generated the three vertebrate paralogs and a fourth gene that was quickly lost [3], [10], [28]. Mammals have one *Hh* gene in each of the three subgroups but, as shown later, only *Shh* and *Ihh* genes are found on publicly available avian genomes. Due to the fish-specific genome duplication (FSGD), up to four or five *Hh* genes, *Dhh, Ihha, Ihhb, Shha* and *Shhb*, can be found in teleost species [29]–[32], and a duplicated *Dhh* gene is also present in the genome of *Xenopus laevis,* but not *Xenopus tropicalis*, since all but one *Xenopus* species are allopolyploid [10], [33]. In addition, two *Hh* paralogs are found in the cyclostomes *Lampreta fluviatilis* and *Petromyzon marinus,* which cluster with the *Shh/Ihh* vertebrate group, suggesting that cyclostomes once had a *Dhh* gene but lost it [34] and that the *Shh, Ihh* and *Dhh* members of the *Hh* are more ancient than agnathans. However, the urochordate *Ciona intestinalis* has two *Hh* genes, *CiHh1* and *CiHh2*, that cluster with the invertebrate *Hh* group and are likely to result from a lineage-specific duplication [35].

Studies of adaptive evolution in invertebrate members of the *Hh* family showed evidence of positive selection, with different rates in each of the two domains encompassing the Hh proteins, a pattern which appears to be related with the divergence of the two main bilaterian groups Ecdysozoa and Deuterostomia [36]. Since duplicated genes often diverge functionally and vertebrate *Hh* paralogs have distinct physiological roles, the study of adaptation in the vertebrate members of the *Hh* family could provide valuable insights into the evolutionary forces acting on vertebrate *Hh* genes and the distinct functional roles of their coded proteins.

The increased availability of sequenced vertebrate genomes and resolved tridimensional structures, has facilitated the study of adaptive evolution. The main goal of this study was to assess the adaptive evolution of *Hh* genes in all vertebrates using a comparative genomics framework at two levels. First, we studied the synteny of vertebrate *Dhh* genes compared to the other two vertebrate *Hh* paralogs, to retrace their evolutionary history after the two rounds of whole genome duplication but also to search for Dhh-coding sequences on avian genomes. Second, we evaluated signatures of positive and negative selection using gene- and protein-level approaches. Due to the small number of non-avian and avian reptile genomes in public databases and to the reduced information on the *Hh* gene family in these lineages, we used a set of recently sequenced extensive dataset of 45 avian and three non-avian reptilian genomes (Zhang et al. in preparation; Jarvis et al. in preparation; Green et al. in preparation), and characterized this gene family in these species. We show that vertebrate *Hh*-paralogous genes evolved from conserved duplicated large-scale chromosomal linkage groups. With non-avian and avian reptile lineages, we confirmed that most birds seem to have all three *Hh* paralogs, contrasting with the publically available genomic data where the *Dhh* paralog was missing. As observed in previous works, signatures of selection varied between vertebrate Hh-protein domains. However, we also found that after duplications the three vertebrate members of this family evolved under different evolutionary rates, mainly within the Hog domain. Adaptive evolutionary analyses at the protein-level showed evidence of positive selection across the two main domains that comprise vertebrate *Hh* proteins, mainly at the protein surface.

## Results

### Evolution at the Genomic Level

**Figure 2:**
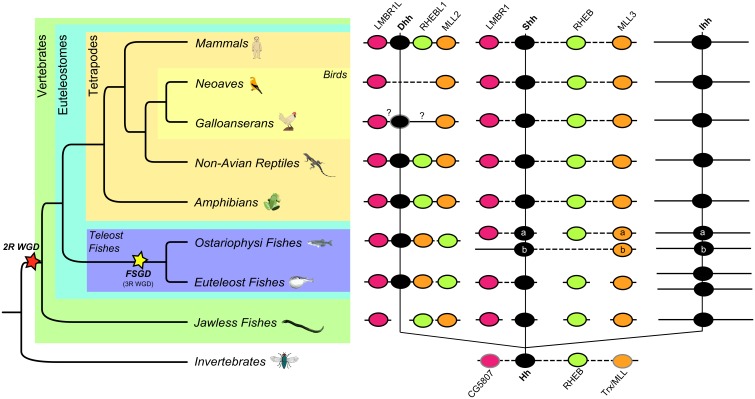
Illustrative representation of the presence of *Hh* and syntenic related genes in vertebrates according to Genomicus 64.01 [37] and the GenBank [42] and Ensembl [38] databases. Information for all vertebrate and invertebrate species listed on Genomicus 64.01 [37]. A dotted line between two genes is equivalent to a gap in the alignment, i.e. the two genes are neighbors in this species but not in the reference species, where their orthologs are separated by one or more genes. On the other hand, a large white space indicates that the genes are found on the subject genome but are located on different chromosomes/scaffolds. A question mark (?) indicates that the syntenic relationship is not known. Genes outlined by a black line where found using Genomicus 64.01 [38] and genes outlined by a grey line were found by blast searches over the GenBank [42] and Ensembl [38] databases. The absence of a gene represents that that gene is not annotated on Genomicus 64.01 and was not found by blast searches.

To evaluate why the *Dhh* paralog is missing in the current publically available avian genomic databases, we determined the synteny of the *Dhh* gene in the genomes of several species that represent major groups of vertebrates using the GENOMICUS v64.01 database browser [37] and 45 avian and three non-avian reptilian species provided by the BGI-G10K avian Phylogenomics Project (Zhang et al.; Jarvis et al. manuscripts in preparation) (Fig. 2). We observed that the *Dhh* gene formed a conserved linkage group with the *LMBR1L, RHEBL1* and *MLL2* genes in all the tetrapods in the database. Teleost fishes had a similar group composed of the *LMBR1L*, *Dhh* and *MLL2* genes, where the *Dhh* and *MLL2* genes are adjacent to each other and the *RHEBL1* gene is more-separated.

**Figure 3:**
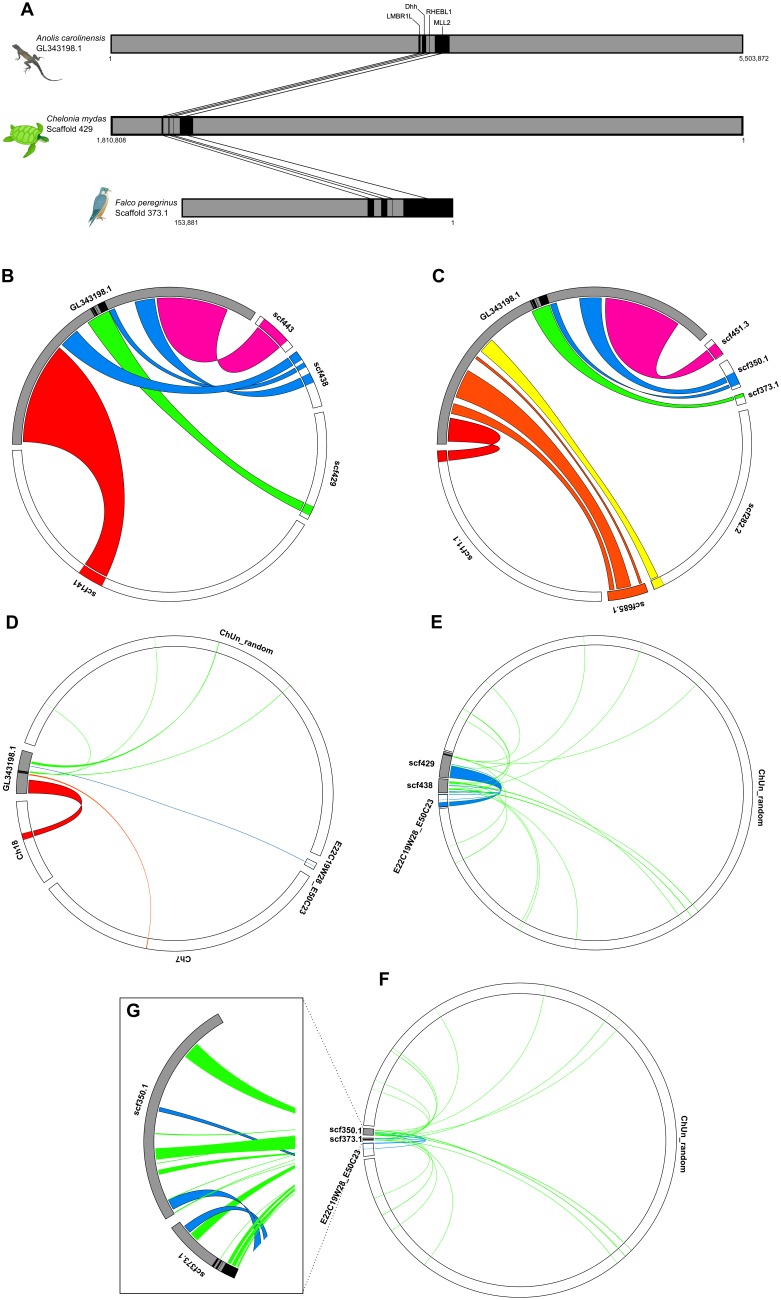
Homology between the *Anolis carolinensis Dhh* gene synteny and the *Chelonia mydas, Falcon peregrinus* and *Gallus gallus* genomes, using nucleotide information. (A) The tetrapod *LMBR1L–Dhh-RHEBL1-MLL2* gene cluster is found on the scaffold GL343198.1 scaffold of the *A. carolinensis* assembly (anoCar 2.0 [105]), and a similar cluster is also found on the 373.1 scaffold of the *F. peregrinus* genome assembly. (B) The four main *C. mydas* scaffolds show great homology for specific regions of the lizard GL343198.1 scaffold, (C) six on the *F. peregrinus* genome, and (d) on *G. Gallus* genome homology is found on two macrochromosomes, a linkage group and the Un_random chromosome. (e) The 429 and 439 *C. mydas* scaffold have high homology with a great part of the chicken E22C19W28_E50C23 linkage group and several random regions of the chicken Un_random chromosome. (F) The 350.1 and 373.1 *F. peregrinus*scaffold have high homology with several random regions of the *G. gallus* Un_random chromosome. (E) Hits for the *F. peregrinus* cluster are found on *G. gallus* genome mainly for the *MLL2* gene.

Using solely data from previous databases, only the *LMBR1L* and *MLL2* genes were found in the genome of a songbird, the zebra finch, a Neoaves species (*Taeniopygia gutatta*). In Galloanserae (*Gallus gallus* and *Meleagris gallopavo*) only *MLL2* was annotated (Fig. 2) but BLAST (TBLASTN and BLASTp) searches of the 45 avian and non-avian reptilian genomes showed evidence of all the genes that comprise this cluster in this tetrapod lineage (Table S9 in S1 Tables). The best results were obtained from the genomes of the Neoavian species *Falco peregrinus* (peregrine falcon), *Melopsittacus undulatus* (budgerigar) and *Haliaeetus leucocephalus* (bald eagle) and the two non-avian reptiles, *Alligator mississippiensis* (american alligator) and *Chelonia mydas* (green turtle) (Table S9 in S1 Tables). The conserved tetrapod *LMBR1L–Dhh-RHEBL1-MLL2* linkage group was intact in *F. peregrinus* and *C. mydas* (Fig. 3A) while in *M. ondulatus, H. leucocephalus* and *A. mississippiensis,* all four genes were identified, but were dispersed on small scaffolds.

Comparing the synteny of the *Dhh* gene in the lizard (*A. carolinensis*) and turtle (*C. mydas*) with the birds *F. peregrinus* and *G. gallus* by BLAST searches (S1 Material), we found four main turtle scaffolds and six falcon scaffolds that had high similarity with specific regions of the lizard GL343198.1 scaffold (Fig. 3B and C), while in the chicken genome we found similarity with regions of two macrochromosomes, a linkage group and the Un_random chromosome (Fig. 3C). Although the correspondence among the genomes was clear for the portions of the lizard GL343198.1 scaffold that are outside the region of *Dhh* synteny, it was more difficult to discern clear correspondence within the *Dhh* and syntenic regions (Fig. 3B, C and D). This is likely because upstream of the *LMBR1L* gene on the lizard scaffold there are three genes that are members of the Tubulin-α family [37], [38], a highly conserved and gene-rich family coding for an important structural family of proteins [39], [40]. As expected, we found matches beyond this region on a turtle scaffold (429) and falcon scaffold (373.1), without dispersed matches. In the chicken WUGSC2.1 assembly the best matches were with the partially-assembled Un_random chromosome, with many highly dispersed hits representing small portions of the genes composing the linkage group (Fig. 3B, C and D).

In *F. peregrinus,* the 373.1 scaffold had high similarity with several random regions of the *G. gallus* Un_random chromosome and the E22C19w28_E50C23 linkage group, which also matched portions of the turtle scaffolds 429 and 438 (Fig. 3E and F). Similarly, the *F. peregrinus* 350.1 scaffold had regions with high similarity with random positions on the *G. gallus* Un_random chromosome, as well as with two regions of the *G. gallus* E22C19w28_E50C23 linkage group, one located near the 373.1 scaffold hit. On the other hand, when we looked at the region of the 373.1 scaffold where the *Dhh* and syntenic genes were found on the falcon genome (Fig. 3G), we found matches in the *G. gallus* genome only for the falcon *MLL2* gene, which is highly dispersed on the chicken Un_random chromosome.

To infer if a similar linkage group is also common among the other *Hh* paralogs, we searched for paralogs of *LMBR1L, RHEBL1* and *MLL2* associated with the *Shh, Ihh* and *Hh* genes (Fig. 2). Such paralogs were found on the same chromosome/scaffold as the *Shh* gene in the genome of all tetrapod species studied but none were near the *Ihh* gene (Fig. 2 and Table S9 in S1 Tables). *LMBR1, RHEB* and *MLL3* were located alongside of *Shh* and in the same relative order across all tetrapods as is also observed in the *LMBR1L–Dhh-RHEBL1-MLL2* linkage group. However, these genes were separated by several other interspersed genes, which together formed a high-dimension linkage group that was present in at least all tetrapods.

Teleost fishes had a similar linkage group (Fig. 2), with the *Shh* and *LMBR1* genes located together on the same chromosome/scaffold. However, in this case, *RHEB* and *MLL3* were located elsewhere. *Danio rerio* (zebra fish, the only available representative of an ostariophysi fish), has a duplication of the *Shh* gene (*Shha* and *Shhb)* [29] and we observed that *Shha* and *LMBR1* were on the same chromosome but were separated from *RHEB* and *MLL3*. *MLL3* is also duplicated in the genome of teleost fishes [37], [41], where *MLL3a* is on the same chromosome as *RHEB* and *MLL3b* is on the same chromosome as *Shhb.* However, in the genome of euteleost fishes, the *RHEB* and *MLL3* duplicates were separated (Fig. 2).

We identified all these genes, with the exception of *Dhh* gene as expected [34], in the sea lamprey *Petromyzon marinus*, the only available representative of jawless fishes. However, it was not possible to determine their synteny, as the currently available lamprey assembly (WUGSC v3.0) is more fragmented and each of the genes of interest are found on different small scaffolds (Fig. 2). We observed that one *RHEB* gene has been annotated in the genome of *Drosophila melanogaster* and is located on the same chromosome as *Hh*. *LMBR1* and *MLL2/3* in the *D. melanogaster* genome have not been annotated, but our searches (TBLASTN and BLASTp) of the GenBank [42] and Ensembl [38] databases for *LMBR1* and *MLL2/3* in this invertebrate genome revealed that the *CG5807* matched *LMBR1* genes with about 60% sequence identity [42] and *Trx* matched *MLL* genes with about 50% sequence identity [43], [44], as expected. These genes were found on the same chromosome, co-linear with *Hh* and *RHEB* and in the same order as in the described linkage groups of other species (Fig. 2), but separated by larger gene gaps. These findings suggest the presence of a linkage group in *Drosophila*, which in vertebrates evolved into three different clusters after the two WGD.

### 2. Evolution at the Gene and Protein Level

**Figure 4:**
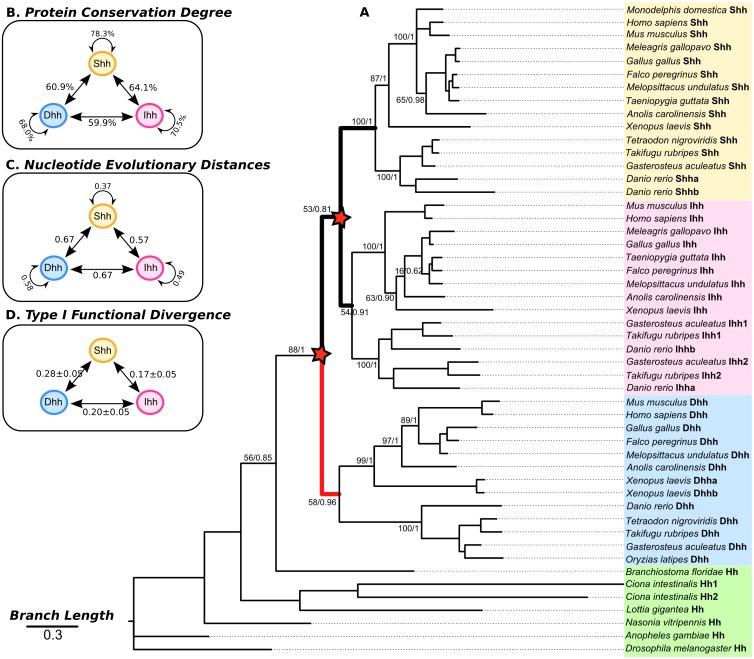
Phylogenetic relationship of *Hh* coding sequences. (**A**) The phylogenetic tree was constructed using Maximum Likelihood (PhyML [83]) and Bayesian inference (MrBayes [84], [85]) algorithms, with supporting values as branch labels (ML/Bayesian). The tree is drawn to scale, with branch lengths in the same units as those of the evolutionary distances used to infer the phylogenetic tree. The post-duplication branches tested with the branch model implemented in PAML [46] are represented in bold and the faster evolving ones are coloured red. (B) The degree of similarity between Hh proteins and (C) the evolutionary distances between *Hh* coding sequences, inferred using MEGA 5 [77], and (D) the type I functional divergence coefficient values (θ_I_) between Hh proteins, inferred using DIVERGE 2.0 [60], are shown.

After identifying all three homologs in some avian species, we were able make comparisons of protein coding sequence evolution across all three vertebrate lineages. At the nucleotide coding-sequence level, the three vertebrate *Hh* paralog genes *(Shh, Ihh, Dhh*) are conserved [45] in all vertebrate species studied, with 0.57 substitutions per site between *Shh* and *Ihh* and 0.67 between *Ihh* and *Dhh* and also 0.67 between *Dhh* and *Shh* (Fig. 4C). This pattern was also observed at the protein level, with 64.1% sequence identity between Shh and Ihh proteins, 59.91% between Ihh and Dhh and 60.9% between Dhh and Shh (Fig. 4B). Within each group, the sequences also had different substitution and identity rates, with *Shh* showing the highest, and *Dhh* the lowest values (Fig. 4B and C). The invertebrate *Hh* genes used as outgroups had a mean of 0.87 substitutions per site within *Hh* coding sequences and a mean of 0.84 among *Hh* coding sequences and *Dhh, Ihh* and *Shh*. At the protein level, the *Hh* sequences had 54.0% similarity with each other and a mean of 54.65% with *Dhh*, *Ihh* and *Shh* proteins. However, these results are not significant because the represented outgroups are highly divergent.

We performed a phylogenetic analysis of *Hh* coding sequences in 50 species and found similar overall topologies with both Bayesian (BY) and Maximum Likelihood (ML) methods. In agreement with previous works [3], [10], [28], the two phylogenetic methods retrieved the (Hh, (Dhh, (Ihh, Shh))) topology (Fig. 4A). The three vertebrate Hh-paralog groups were highly conserved but after duplications the *Shh* group experienced more constrained evolution, while Ihh and Dhh were more divergent and thus apparently evolved under increasingly relaxed constraints. To better understand the forces influencing this gene family evolution we used this estimated phylogenetic tree to test for selection signatures and functional divergence in the vertebrate members of the Hedgehog family.

#### 2.1 Selective constraints at the nucleotide codon-level after Hh genes duplications

To test for different evolutionary rates upon duplication, we first assessed positive selection on post-duplication branches using the branch models implemented in PAML v4.03 [46]. The likelihood ratio test (LRT) between the alternate and null model likelihoods showed that only the *Dhh* branch fit the two-ratio model (Table 1), suggesting that this branch has evolved at a different rate than the other three. When checked for evidence of positive selection [47], the null hypothesis was not rejected, suggesting that this branch has not been under positive selection but instead has evolved under relaxed selective constraints (Table 1). However, this lineage-base analysis assumes that all amino acid coding sites have experienced the same selective pressure and since many sites can be evolving at a different rate, it is a very conservative test for adaptive evolution [48]. Thus, we used the site models implemented in PAML to test for signatures of adaptive evolution across the *Dhh, Ihh* and *Shh* coding sequences (Table 2) and found that they had overall ω values of 0.114, 0.080 and 0.058, respectively, with no positively selected residues. In all cases the M3 and M7 models were accepted, which signifies that each codon on *Dhh, Ihh* and *Shh* sequences were under variable selective pressures, with no evidence of positive selection.

**Table 1:** Likelihood parameter estimates under lineage-specific model of post-duplication branches of *Hh* vertebrate paralogs, branch calculated with PAML v4.3 [51].

Model		ω_0_	ω_1_	*Lnl*	Models compared	LRT (2Δl)	p-value	*df*
**A**	One-ratio (M0)	0.0610	NA	−29435.14				
**B**	Dhh two-ratio (unconstrained)	0.0610	999	−29432.95	A and B	4.38	0.04	1
**C**	Dhh two-ratio (constrained)	0.0610	1	−29433.13	C and B	0.36	0.55	1
**D**	Ihh/Shh two-ratio	0.0612	921	−29434.62	A and D	1.05	0.31	1
**E**	Ihh two-ratio	0.0610	0.8302	−29434.96	A and E	0.37	0.54	1
**F**	Shh two-ratio	0.0610	0.1115	−29434.85	A and F	0.59	0.44	1

**Table 2:** Likelihood parameter estimates under site-specific models of *Hh* vertebrate paralogs, branch calculated with PAML v4.3 [51].

Gene	Model	Parameters	*Lnl*	Models Compared	LRT (2Δl)	p-value	*df*
*Dhh*	M0	ω0 = 0.08479	−7360.011	M0 *vs* M3*	190.185	0.000	4
	M3	ω0 = 0.00492 ω1 = 0.08337 ω2 = 0.31612	−7162.160				
		p0 = 0.39785 p1 = 0.34084 p2 = 0.26131					
	M7	p = 0.41513 q = 3.11107	−7163.063	M7* *vs* M8	0.006	0.997	2
		ω = 0.114					
	M8	p0 = 0.99999 p = 0.41486 q = 3.10767	−7163.066				
		(p1 = 0.00001) ω1 = 2.90725					
*Ihh*	M0	ω0 = 0.06236	−8206.454	M0 *vs* M3*	740.516	0.000	4
	M3	ω0 = 0.00520 ω1 = 0.11825 ω2 = 0.34903	−7836.196				
		p0 = 0.59836 p1 = 0.27289 p2 = 0.12874					
	M7	p = 0.25686 q = 2.77341	−7838.824	M7* *vs* M8	0.002	0.999	
		ω = 0.080					
	M8	p0 = 0.99999 p = 0.25687 q = 2.77361	−7838.825				
		(p1 = 0.00001) ω1 = 1.00000					
*Shh*	M0	ω0 = 0.04658	−7648.861	M0 *vs* M3*	612.130	0.000	4
	M3	ω0 = 0.00188 ω1 = 0.06952 ω2 = 0.26493	−7342.796				
		p0 = 0.60204 p1 = 0.25287 p2 = 0.14509					
	M7	p = 0.20836 q = 3.15101	−7342.669	M7* *vs* M8	0.007	0.996	2
		ω = 0.058					
	M8	p0 = 0.99999 p = 0.20836 q = 3.15102	−7342.672				
		(p1 = 0.00001) ω1 = 5.18630					

An asterisk (*) marks the accepted model.

**Figure 5:**
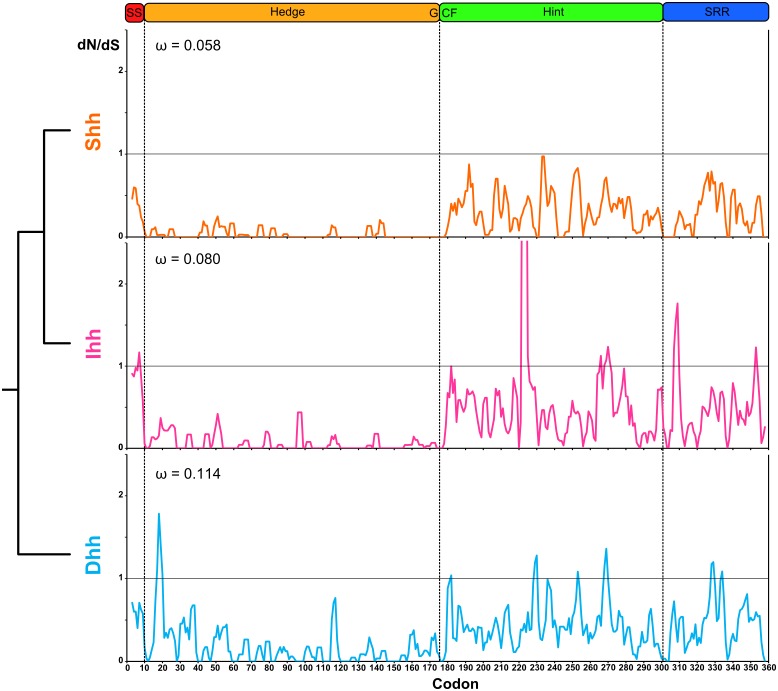
Differences in the selection pattern of the three vertebrate *Hh* paralogs. Sliding windown analysis of the dN/dS ratio applying the SLAC and FEL methods [49] for the three vertebrate *Hh* paralogs, represented as a mobile mean with a period of 3. The phylogenetic relationship between each group and the mean omega value (ω) for each branch calculated with PAML v4.3 [46] are shown. The Hh proteins domains are displayed as annotated for the Hh, Dhh, Ihh and Shh proteins on the GenBank [42] and UniProt [113] databases.

As our data do not show evidence of positive selection, we used the Single Likelihood Ancestor Counting (SLAC) and the Fixed Effects Likelihood (FEL) methods [49], as implemented in the Datamonkey web server [50], [51], to test for evidence of purifying selection and to identify the corresponding residues. In agreement with our previous results, no evidence of positive selection was observed for the three *Hh* paralogs in all species with either method [49] (Table S1 in S1 Tables). With a significance threshold of 0.05 (P<0.05), both methods did not identify any residues under positive selection but found a large percent of residues under negative selection. Being less-conservative and more-powerful, the FEL method was able to find more residues under negative selection than the SLAC model [50]: the SLAC model showed that for the Dhh, Ihh and Shh proteins, 28%, 39% and 38% of the residues experienced negative selection while the FEL method detected patterns of negative selection at 45%, 54% and 55% of the residues for each respective paralog (Table S1 in S1 Tables). Although, both methods identified codons with dN/dS >1 (Fig. 5), none were statistically significant (Table S2 in S1 Tables). With a relaxed significance threshold of 0.10, these codons were still not positively-selected and, as expected, the number of negatively selected codons increased (Table S1 in S1 Tables). Despite the lack of high dN/dS, we found that among the four main functional regions of the three vertebrate *Hh* gene coding sequences, most of the codons with stronger purifying signatures were located in the Hedge/signaling domain (Fig. 5). Values for each codon were lowest for *Shh* and highest for *Dhh*.

#### 2.2 Selective constraints at the amino acid-level after Hh genes duplications

**Figure 6:**
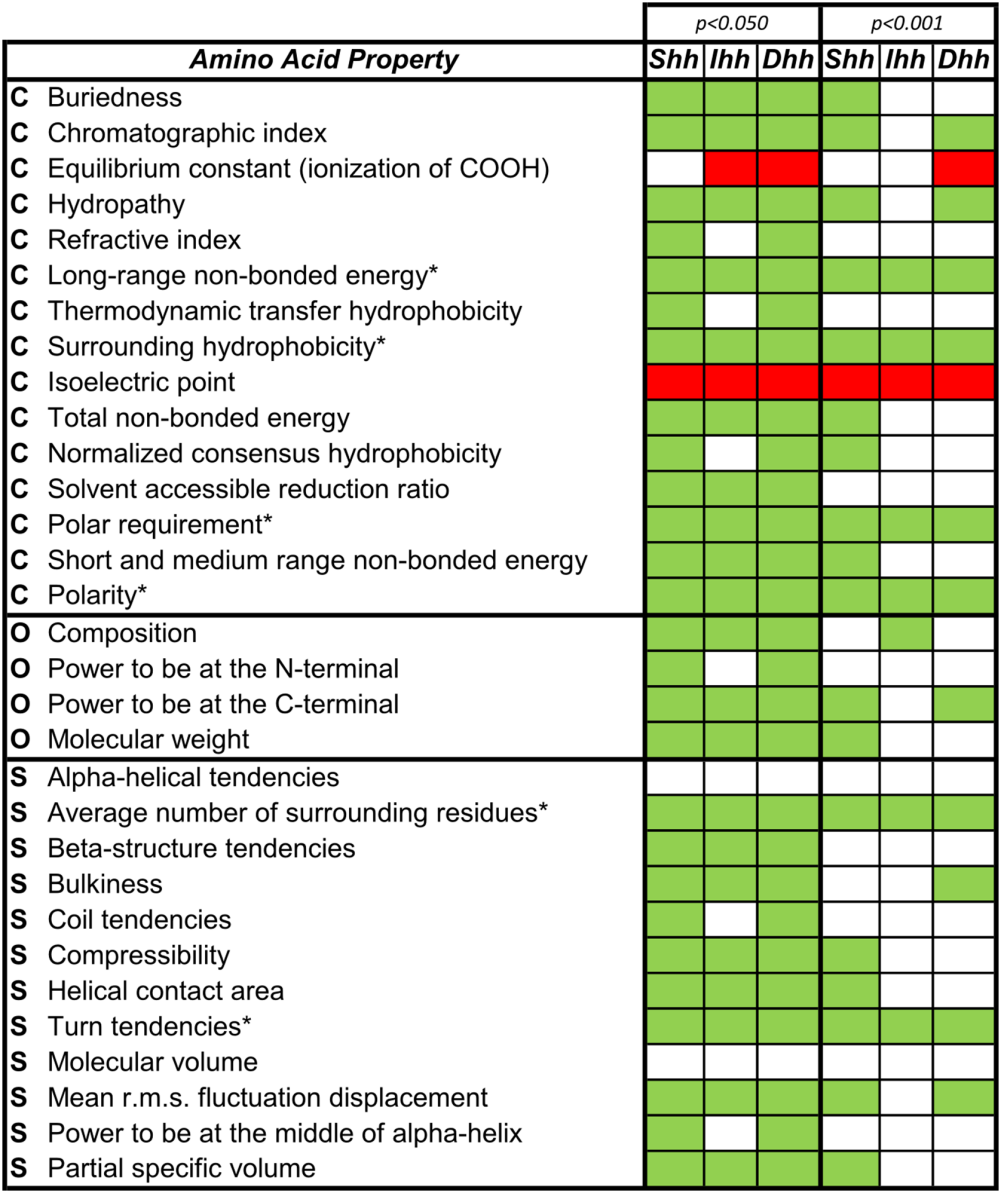
Amino acid properties under positive (red) and negative (green) selection in vertebrate *Hh* coding sequences. Two different signifcance levels are shown: (Z-score > |1.64|) to detect significant selective signatures and (Z-score > |3.09|) to detect strong selective signatures. Amino acid properties are classified as chemical (C), structural (S) and other (O), according to da Fonseca *et al.* [54].

**Figure 7:**
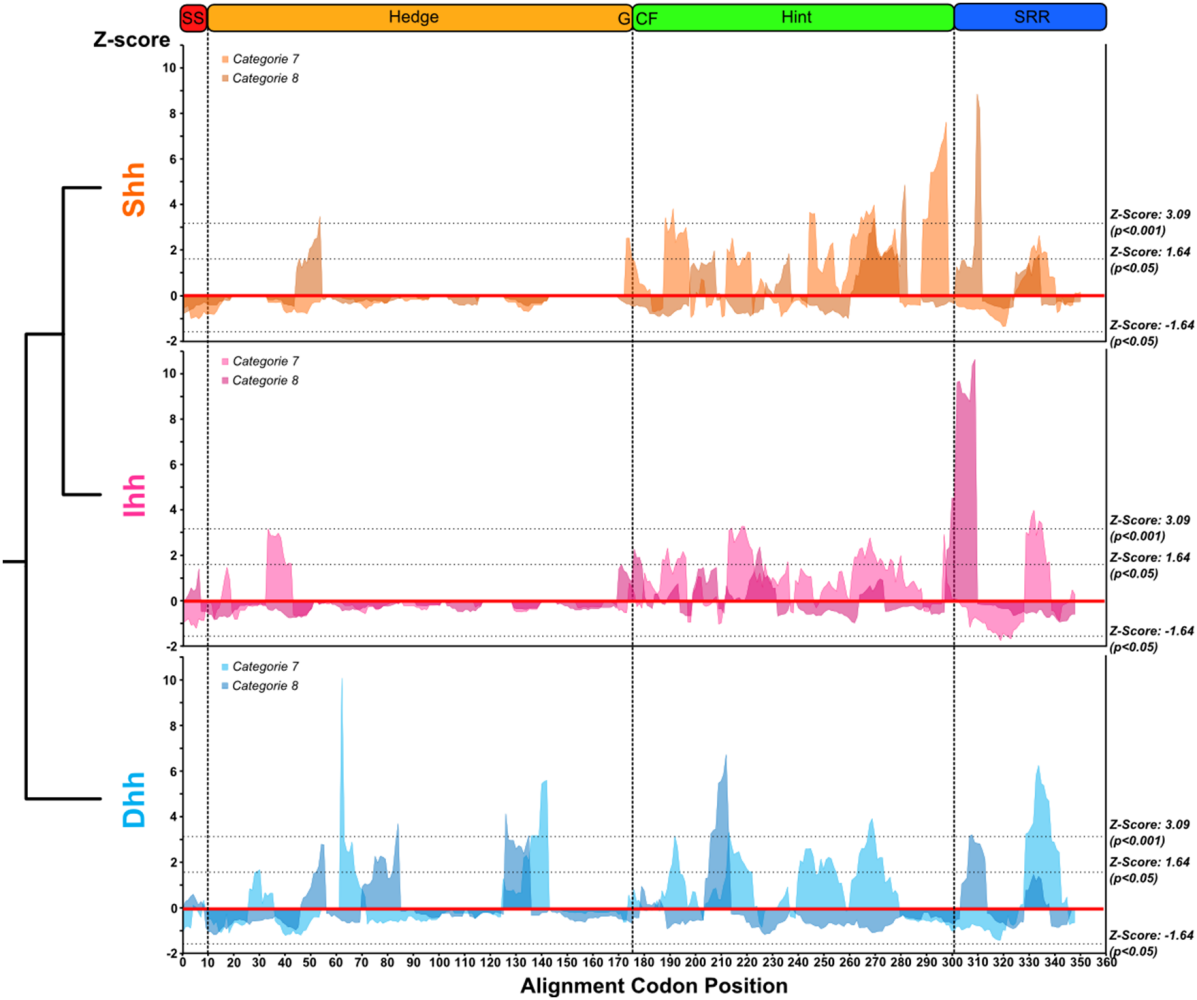
Differences on the amino acid isoelectric point property selection pattern for the three vertebrate *Hh* paralogs. Sliding window analysis for the Z-scores calculated for categories 7 and 8 using TreeSAAP [56] for the three vertebrate *Hh* paralogs, showing the phylogenetic relationship between each group.

Concerns have been raised over the utility of the site selection models that use ω ratios to detect subtle molecular adaptations, as they can fail to detect positively and negatively selected sites evolving under possible biochemical constrains [52]–[55]. Therefore, we used TreeSAAP [56] to detect evidence of positive and negative selection across destabilizing substitutions to infer the biochemical forces acting on the evolution of Hh genes and to compare diversification patterns of vertebrate *Hh* proteins relative to invertebrates. We started by assessing which destabilizing properties were under negative and positive selection in each of the three vertebrate *Hh* paralogs and found that 24 of 31 biochemical properties were under negative selection, of which six were under strong purifying selection (Fig. 6). These negatively-selected properties are classified as having both chemical and structural properties, highlighting the importance of the chemical and structural features of *Hh* proteins in the activation of the *Hh* signaling pathway. Interestingly, one property, the amino acid isoeletric point, was under strong positive selection in all paralogs. This chemical property correlates with the pH at which the amino acid surface carries no net electrical charge, suggesting that this may be an adaptive feature of the vertebrate *Hh* paralogs. The sliding window analysis of amino acid isoelectic property Z-scores (Fig. 7) showed strong positive selection (P<0.001) in the Hog domain and to a lesser extent over the Hedge domain (P<0.05).

The amino acid isoelectric point was positively selected in all vertebrate *Hh* paralogs, but in different regions for each paralog (Fig. 7). Across the Hedge domain alignment, codon positions were under positive selection from positions 33–55 for the three paralogs, including between positions 33–44 was in the *Ihh* group, and positions 44–55 in the *Shh* and *Dhh* paralogs. Two other regions in the *Dhh* Hedge domain were under positive selection for the amino acid isoelectric point: one between positions 62 and 84 and other from 126–142. In the Hog domain, 7 regions were under positive selection for this property: 5 on the Hint module and 2 on the SRR. Of these, only two regions of the Hint module were not common to the three paralogs: one from positions 240–260 was common to *Shh* and *Dhh* and another from 285–296 was unique to *Shh*.

Different patterns of amino acid properties selection were also observed within paralogs at different significance thresholds (Fig. 6). At a significance of 0.05, the same number of negatively selected properties was found for both *Shh* and *Dhh* paralogs, but a reduced number was found for *Ihh*. In addition, the amino acid equilibrium constant (ionization of COOH) was under positive selection for the *Ihh* and *Dhh* paralogs. When we reduced the significance threshold to 0.001, despite the common properties described above, other properties were under strong negative-selection within different paralogs. As expected from the nucleotide codon-level analyses, a higher number of amino acid properties were under strong negative selection in the Shh proteins. The higher number of strongly negatively-selected properties for *Dhh* than *Ihh* was unexpected, as *Ihh* showed stronger purifying signatures at the nucleotide codon level. However, the amino acid equilibrium constant was under strong positive selection for *Dhh*, which was not observed for the other two paralogs at this threshold. Despite these differences, the majority of these strong negatively-selected changes corresponded to altered chemical properties.

At the amino acid level, we found 20 strongly positive-selected positions in the *Shh* group for at least one amino acid property, while for *Ihh* and *Dhh* there were 27 and 32 sites, respectively (Table S3 in S1 Tables). The majority of these were located in the Hog domain, as expected, and many of them comprised sites that at a codon level had dN/dS >1 but were not statistically detected to be under positive selection (Table S2 and Table S3 in S1 Tables). However, some of the positively selected sites detected by TreeSAAP were identified to be under negative selection with FEL (Table S1 and Table S3 in S1 Tables). From the codon and amino acid alignments these positions corresponded to variable sites with a high rate of non-synonymous substitutions, surrounded by highly conserved positions, suggesting that FEL overestimated the dS value for these positions because of their very negatively-selected environment. When we applied the empirical threshold of at least three properties showing signatures of positive selection, the number of positively selected residues decreased to 8 in *Dhh*, 3 in *Ihh* and 1 in *Shh*. These were only found in the Hog domain and none of them were previously identified as being under negative selection at the codon level. However, 3 in *Dhh* and 1 in *Ihh* had ω values above 1 (Table S3 in S1 Tables). Notably, *Shh* residue 385 (numbered according to the *Homo sapiens* sequence) showed positive selection in 7 amino acid properties and was the only positively selected residue identified in this paralog. No homologous residues to this one were found in the alignment of the *Dhh* and *Ihh* paralogs, but positions surrounding this position shared the same signatures of positive selection within both paralogs: residue 358 for *Dhh* and residues 372 and 373 for *Ihh* (Table S3 in S1 Tables). In addition, despite being located at some distance from these residues on the *Dhh* protein sequence, the *Dhh* residue 396 also had 7 amino acid properties under positive selection.

#### 2.3 Functional divergence of Hh proteins after duplication

After gene duplication, the historic pattern appears to have been that one *Hh* gene copy maintained most of the original function while the other copies accumulated changes leading to functional diversification, with different *Hh* paralogs and selection signatures. Therefore, we tested our data for the prevalence of type I and type II functional divergence using DIVERGE 2.0 [57]–[60]. We found statistically significant changes for type I functional divergence, but not type II divergences. Given the topology presented in Fig. 4, the ML estimate of the coefficient (θ_I_) of type I functional divergence lowest between *Shh* and *Ihh*, intermediate between *Ihh* and *Dhh*, and highest between *Shh* and *Dhh* (Fig. 4D and Table S4 in S1 Tables).

**Figure 8:**
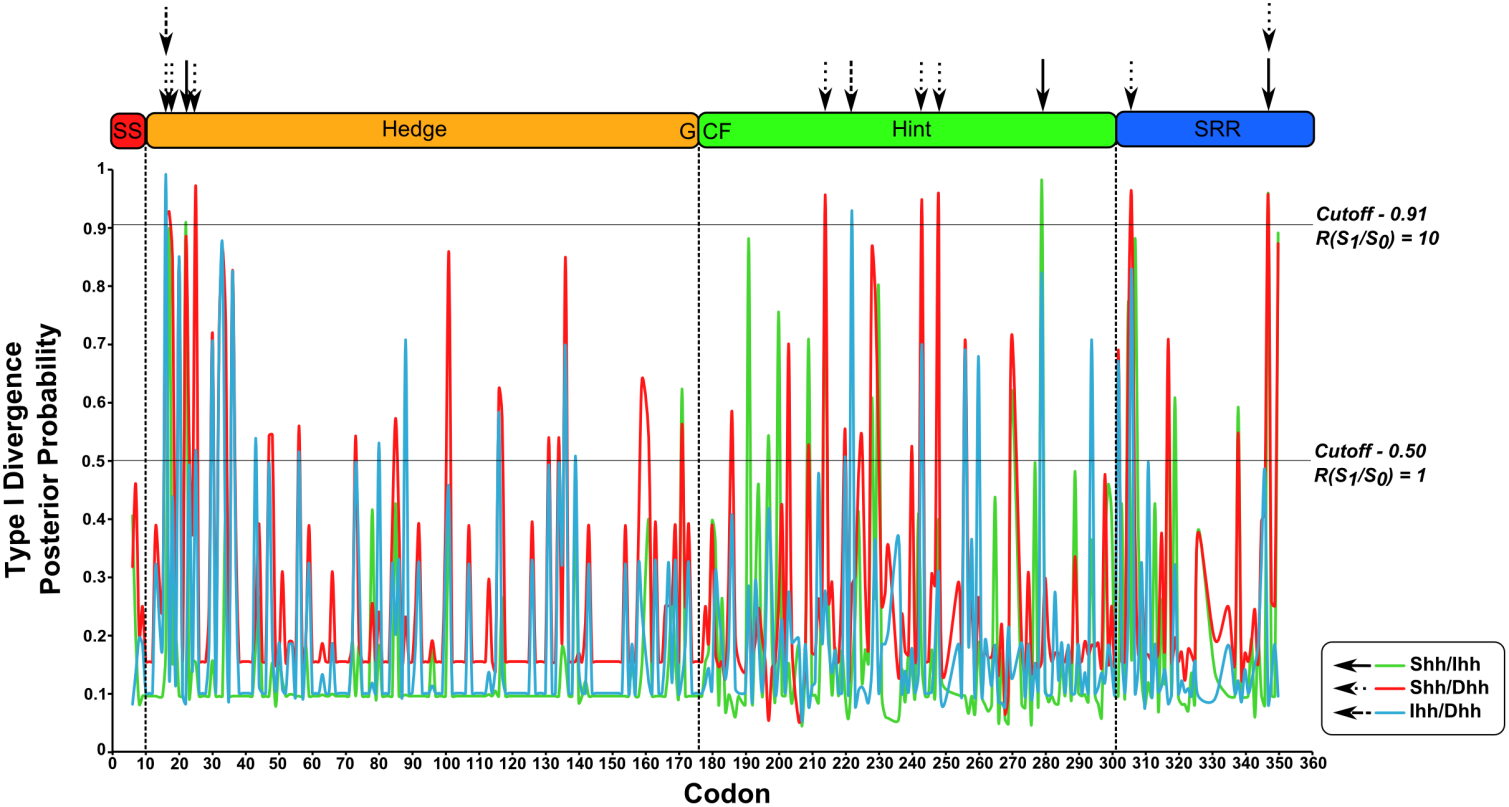
Type I functional divergence over the vertebrate Hh paralogs. Posterior probability for predicting critical amino acid residues for the functional divergence between the three vertebrate members of the Hh family. The arrows point to the residues with P(S_1_|S_0_) >0.91 and their position on the Hh proteins primary structure.

The site-specific profile based on the posterior analysis for scoring amino acid residues that are likely to be involved in type I functional divergence between vertebrate *Hh* paralogs is presented in Fig. 8, and shows that the higher posterior probabilities are found within the Hog domain. Between *Shh* and *Ihh*, 18 of 358 sites were above a posterior probability of 0.5, and that number increased to 46 between *Shh* and *Dhh* and to 19 comparing *Ihh* and *Dhh* (Fig. 8). Using the cutoff of 0.91 (corresponding to a posterior odd ratio R(S_1_|S_0_) >10), we identified 3 sites that significantly accounted for the type I functional divergence between *Shh* and *Ihh*, 8 between *Shh* and *Dhh* and 2 between *Ihh* and *Dhh* (Table S5 in S1 Tables and Fig. 8). These predicted functional sites were not equally distributed throughout their respective proteins, but clustered in the N-terminal region of the Hedge domain and in the Hint and SRR regions. Different clusters were found for different pairs of paralogs (Fig. 8) and those across the Hog domain were in regions that were positively selected for the amino acid isoelectric point property (Fig. 7 and Fig. 8).

We observed 3 sites with a posterior probability above 0.91 that were responsible for type I functional divergence between *Shh* and *Ihh* and that were highly conserved in *Shh* proteins but highly variable in *Ihh* proteins. The same was observed for the *Shh*/*Dhh* pair, but not for the *Ihh*/*Dhh* pair (S1 Fig.). In addition, 2 of the type I functionally divergent sites between *Shh* and *Ihh* and 5 found between *Shh* and *Dhh* corresponded to negatively selected residues (Table S3 and Table S5 in S1 Tables). Thus, these residues accounted for the functional divergence of Shh proteins relatively to the other two paralogs.

#### 2.4 Structural analysis of selected domains

**Figure 9:**
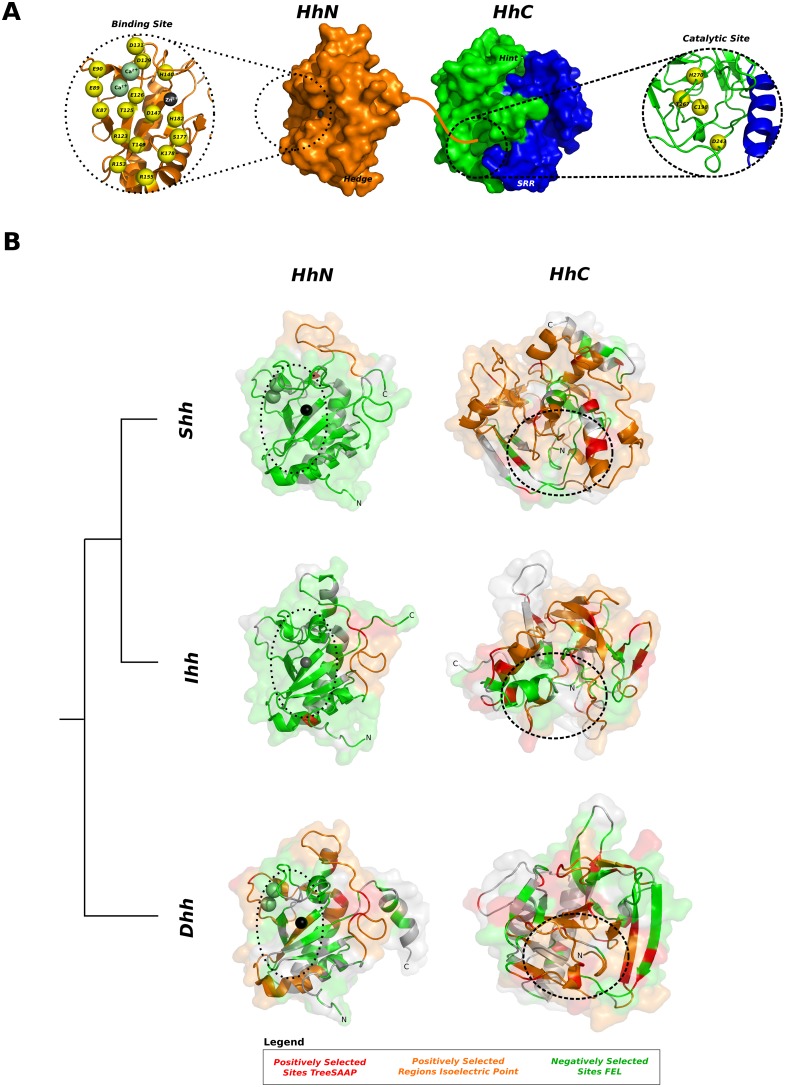
Tridimensional arrangement of negatively and positively regions in the Hedge domain of vertebrate Hedgehog proteins. (A) Tridimensional representation of Hedge (PDB: 3HO5) and Hog (Shh modelled by homology) domains, coloured according to key identified regions. A straight orange line denotes how both domains may be linked in the pro-protein. Key residues important for binding and forming the catalytic site are represented in yellow spheres, numbered according to the human Shh protein [72], [113], [114]. (B) Tridimensional arrangement of negatively and positively selected regions across the Hedge (HhN) and Hog (HhC) domains. Proteins (ShhN: PDB 3HO5, DhhN: PDB 2WFR; IhhN: PDB 3K7G) represented in grey cartoon with transparent surface. Calcium ions are not represented on the IhhN peptide due to the absence of the ions on the PDB file. Negatively selected sites (green) identified with FEL, positively selected regions for the amino acid isoelectric point property (orange) and positively selected sites (red) identified with TreeSAAP are shown for each paralog domain. A dashed circle denotes the position of the zinc/calcium binding site and the catalytic site.

To further relate the spatial position of the regions under selection and the divergent sites on the tridimensional structure of the three vertebrate Hh paralog proteins, it was necessary to assess them relative to the tridimensional structure of the *Shh*, *Ihh* and *Dhh* proteins. We started by mapping the negatively-selected sites identified at the codon level on the tridimensional structure of Hedge domain for each paralog and observed that they are evenly distributed on the interior and on the surface of the HhN peptide (Fig. 9 and S2 Fig.). Given that the function and folding of proteins are affected by the interactions each residue can form on the surface and core of the tridimensional structure, they can be significantly affected by mutation. Therefore, the finding of such strong negative selection suggests the action of strong constraints that keep the function and the tridimensional structure of the globular signaling peptide unchanged. As the number of negatively selected sites decreased from *Shh* to *Ihh* to *Dhh*, there were fewer sites that were exposed on the peptide surface. This would preserve the interior of the peptide and the zinc and calcium binding cleft, which are important for the interactions of Hedge peptides with receptor proteins (reviewed in [61]).

The regions that were under positive selection based on the amino acid isoelectric point property of the Hedge peptide were located on the surface of the signaling peptides at locations specific to each paralog (Fig. 9 and S2 Fig.). For *Shh* and *Ihh* signaling peptides, the two identified regions comprised a large surface loop of the Hedge signaling peptides close to the binding site. However, both defined different parts of this loop, forming two distinct regions in highly negatively-selected areas and that may provide different adaptive features for these two lineages. With the *Dhh* signaling peptide, the same loop was under positive selection, grouping the two regions that define *Shh* and *Ihh*. However, despite being separate on the primary structure of the *Dhh* signaling peptide, the other two areas previously identified as being under positive selection for this property (Fig. 7) are folded so that the region formed by the positively-selected loop around the binding site is expanded (Fig. 9 and S2 Fig.). Interestingly, the sites identified to be under positive selection for at least one amino acid property are not located in this region.

The tridimensional structure of the Hog domain was predicted for each of the vertebrate Hh paralogs using the *Drosophila melanogaster* HhC peptide as a template. Due to the high divergence among vertebrate Hog sequences, the best three models were similar, but did not superimpose on the spatial space (RMSD: ShhC/DhhC – 2.14 Å; ShhC/IhhC – 2.20Å; IhhC/DhhC – 1.70Å). Interestingly, the catalytic site was always located within a deep pocket on the peptide interior (Fig. 9 and S3 Fig.). When mapped on these models, the negatively-selected sites identified at the codon level comprised residues that were probably located on the interior of the Hog domain and were all arranged around the catalytic site (Fig. 9 and S3 Fig.). The positively-selected regions for the amino acid isoelectric point property were mainly located on the surface of the Hog domain, as were the residues detected as positively selected for at least one amino acid property (Fig. 9 and S3 Fig.). *Dhh* residues 358 and 396, *Ihh* residues 372 and 373 and *Shh* residue 385 were located on the surface around the catalytic site of the Hog domain (Fig. 9 and S3 Fig.), but did not have the same spatial organization in different paralogs. These results suggest, unlike what was observed for the Hedge domain, that purifying constraints only act across the Hog domain to maintain an intact catalytic site, allowing the tridimensional structure of this domain to change under relaxed chemical and structural constrains.

## Discussion

With our discovery of all three members of the *Hh* family in some avian genomes, we confirm and show that all three vertebrate members of the *Hh* family evolved through two rounds of genome duplications in vertebrate ancestors [10]. Our synteny analyses of the *Dhh* gene, compared with the other *Hh* genes, suggested that although the synteny of each of the three vertebrate *Hh* paralogs has remained very conserved, these genes seem to have evolved independently after duplications. This is consistent with the recent finding of an ancestral linkage group that is shared between the amphioux *Hh* and mouse *Hh* genes [62]. Therefore, we hypothesize that before the first round of whole genome duplication, ancestral *Hh* synteny consisted of a conserved linkage group that encompassed at least the ancestral *LMBR, Hh*, *RHEBL* and *MLL* genes. These genes were present in the ancestral vertebrate genome in this same order, but the genes were separated from each other by many other genes and thus formed a high-dimension linkage group.

**Figure 10:**
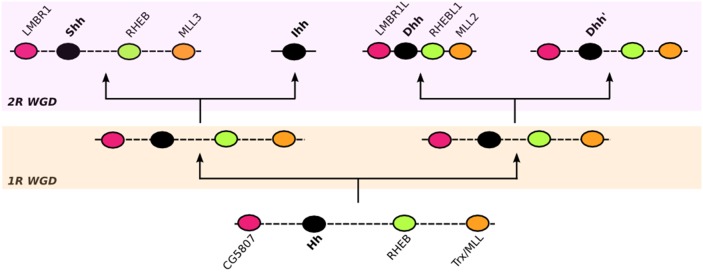
Illustrative representation of the evolution of the LMBR1L–Dhh-RHEBL1-MLL2 linkage group evolution in vertebrates and their paralogs on the synteny of vertebrate *Shh* and *Ihh* genes. A dotted line between two genes is equivalent means these two genes are neighbors in this species but not in the reference species, where their orthologs are separated by one or more genes. The 3^rd^ WGD duplication in the teleost lineage is not represented.

Given these results, we propose (Fig. 10) that after the first duplication, two linkage groups were formed, one including the ancestor of *Shh* and *Ihh* and the other the ancestor of *Dhh*. Before the second round of whole genome duplication, these two clusters may have began to evolve independently, experiencing rearrangements that reduced the size of both linkage groups, but that retained the ancestral duplicated *LMBR, Hh*, *RHEBL* and *MLL* genes. After the second round of duplication, to explain our results, four duplicated linkage groups could have been produced, each with a duplicated version of the more-ancestral conserved linkage group. Further rearrangements before the emergence of vertebrates may have led to the loss of a duplicated *Dhh* gene and to the creation of the synteny currently observed in each of the vertebrate *Hh* paralogs, which has been conserved in the tetrapod lineage, and exists with some further arrangements in the teleostei lineage.

As there is evidence of these linkage group on the genome of all of the studied vertebrate genomes, including jawless fishes, the vertebrate members of the *Hh* gene family would have appeared 600–500 mya, as suggested by Kumar *et al.* [10]. Although it was not possible to study the synteny of the *LMBR, Hh*, *RHEBL* and *MLL* genes in the jawless fish genomes available to-date, the presence of the LMBR1L, RHEBL1 and MLL2 in these genomes is consistent with the hypothesis that cyclostomes diverged after the two WGDs that characterized vertebrate evolution [63]. When we analyzed the teleostei lineage, a different, albeit conserved pattern was found within the two main analyzed classes (ostariophysi and euteleost fishes), suggesting that further rearrangements may have occurred before the third whole genome duplication in early teleosts evolution about 350 MYA [32]. The three conserved syntenic groups seem to have only retained their original order in tetrapods.

We wondered if birds could be an exception, as evidence of genes encompassing the *Dhh* conserved synteny is not found in some of the available avian genomes. However, our comparative genomics analyses using the lizard physical position of this conserved cluster showed that regions neighboring these genes are found in several galliform and neoaves genomes and have been randomly assigned to the Un_Random chromosome of those avian genomes that have mapped karyotypes. The general avian karyotype is composed of 7–9 pairs of macrochromosmes and 30–32 pairs of microchromosomes [64]. Microchromosomes are very small chromosomes that range in size from 3.5 to 23 Mb [65], are remarkably gene rich, have a high recombination rate and consist of a high content on CpG islands [64], which makes them difficult to clone, sequence and distinguish using standard cytogenetic approaches. Thus, genes from many microchromosomes might not have been included in the current avian assemblies but instead represented among the contigs that could not be assigned to a chromosome and have been placed in the virtual Un_Random chromosome [66], [67]. It has previously been demonstrated that sequences arranged on the Un_Random chromosome often can be assigned to small microchromosomes and that many chicken cDNA and EST sequences are absent from avian genome assemblies as well as from the Un_Random fraction, suggesting the absence of large amounts of the corresponding DNA sequences in the chicken genome assembly [68]. Therefore, our hypothesis is that this conserved linkage group may be present in all avian genomes, including galloanserans and neoaves, but that they are probably physically located on one of the microchromosomes. In the *Gallus gallus* linkage group, a good candidate is microchromosome 21, as Trukhina and Smirnov [69] have shown that microsatellites from the linkage group E50C23 are located on this chicken microchromosome.

It would be interesting to perform such comparative genomic analysis for both the *Shh* and *Ihh* syntenies, and compare them with our results. This would provide deeper insights into the genomic evolution of the *Hh* gene family in vertebrates and better uncover the phylogenetic relationships of the vertebrate *Hh* paralogs. Our phylogenetic analysis of vertebrate Hh-coding sequences supported the (Hh, (Dhh, (Ihh, Shh))) phylogenetic topology for the evolution of the vertebrate *Hh* genes, as previously suggested [3], [10], [28]. However, one of our most-significant insights on *Hh* gene family evolution was that strong and variable-purifying selection and type I functional divergence have occurred across the three vertebrate *Hh* branches. *Shh* coding sequences seem to be more conserved than those of *Ihh* and *Dhh* and we showed that each of these vertebrate genes are evolving at very different selective rates, which explains why this phylogenetic relationship is always obtained.

At the codon-level, no evidence of positive selection was identified using the standard PAML models for the three paralogs, although the *Dhh* branch seemed to have been evolving under more relaxed constraints than the other two duplicated branches, in contrast with the other two more slowly-evolving paralogs. Our results suggest that *Shh* coding sequences are evolving with more purifying constraints than the other two vertebrate *Hh* paralogs. This would be consistent with the role of the Shh protein in the pathways of more-complex traits compared the other two members, which is central to the development and patterning of the nervous and skeletal systems and is the most-broadly expressed vertebrate *Hh* member (reviewed in [25]). In contrast, *Ihh* is specifically expressed in only a narrowed number of tissues and *Dhh* is confined to the gonads and narrow regions of the peripheral nervous system and is mainly expressed in combination with *Ihh* [25]. Therefore, mutations affecting the fitness of the *Shh* protein should be more deleterious than those affecting *Ihh*, and *Ihh* changes should be more deleterious than those of *Dhh*. Our results are also consistent with previously published observations that there are stronger purifying constraints on the evolution of genes expressed in early stages of embryonic development, as these mutations will, on average, have more deleterious fitness consequences than mutations in genes expressed later [70], [71]. *Shh* is the first member of the vertebrate *Hh* family to be expressed during vertebrate embryonic development, followed by *Ihh* and *Dhh* [25].

An average of 50% of the vertebrate *Hh* proteins residues were evolving under strong purifying selection and those with the strongest selection signature (most conserved) were located on the N-terminal domain. Most of these residues have been reported to be disease-causing mutational hotspots (Table S1 in S1 Tables). Our results are consistent with two previous studies on the *Hh* gene family where Kumar *et al.* [10] found that in *Drosophila Hh* genes, the Hedge domain coding region had a slower evolutionary rate than the Hog coding region and where Gunbin *et al.* [36] reported that invertebrate members of the *Hh* gene family shared a similar pattern of selective signatures across these two main domains. However, these two papers also suggested that positive selection occurred in the Hint coding region. In contrast, although our codon-level analyses found that evolutionary rates differed among vertebrate *Hh* paralogs and that their distinct regions showed different evolutionary rates, codon models did not identify any significantly positively selected residues in the three vertebrate *Hh* paralogs. Our protein-level analyses were consistent with these two previous works, but provided additional evidence of strong positive selection across the Hog domain and more-relaxed positive selection across the Hedge domain. The Shh members showed lower levels of positive selection and Dhh displayed the strongest signatures of positive selection. Strong purifying selection may be acting on the interior of the two domains that comprise these proteins, both at a chemical and structural level and within both domains, suggesting that strong evolutionary constraints have maintained the core role of the *Hh* proteins.

The signaling activity of the Hedge domain requires a highly conserved tridimensional structure to be recognized by its receptors, as is clear from the highly conserved structure of the HhN peptide among Hh paralogs (Fig. 9) [72]. This may explain why strong negative selection is found both on the interior and on the surface of the signaling peptide. However, being composed primarily of surface-loop regions, positively selected areas found surrounding the ion-binding site may provide adaptive features to the signaling peptide, probably for interactions with different receptor proteins. As different areas of these regions had different patterns of positive selection within vertebrate Hh paralogs, these regions likely facilitate the binding of different *Hh* proteins and receptors. On the other hand, the evidence of more-relaxed conservation and a great amount of positive selection over the Dhh signaling domain was expected from its targeted involvement in the development of gonads and is consistent with previousl reports that genes involved in reproduction and sexual differentiation have higher rates of divergence and positive selection than other genes in the genome, providing reproductive adaptations [73], [74]. Therefore, we hypothesize that a similar link between the physiological signaling role of Dhh in gonadal development and its relaxed evolution may have provided adaptive signaling features during the embryonic development of gonads.

However, negative selection appears to be acting only on the interior of the HhC peptide and mainly around the catalytic site, while positive selection was found on the peptide surface. Our results also suggest that these constraints act only to maintain the catalytic sites and a pocket on the interior of the Hog domain, assuring that the catalytic activity of this domain is retained. It is this domain that provides most of the functional divergence observed among vertebrate *Hh* paralogs. As the sites responsible for this feature are found within areas under positive selection on the surface of the peptide, this divergence may be responsible for their structural features and not for their chemical activity.

## Methods

### 1. Synteny analysis

Synteny analysis was performed using the GENOMICUS v64.01 browser [37], which makes an integration of the data available on the Ensembl database [38] to provide a better visualization of conserved synteny blocks and to reconstruct ancient genomes organization, using the *Homo sapiens* sequences as query. Genes not annotated on the GENOMICUS v64.01 browser [37] were searched on the respective species by TBLASTN and BLASTp over the GenBank [42] and Ensembl [38] databases and mapped localizations were annotated in order to compare it with the localization of putative syntenic genes. Local BLAST databases of 45 avian and three non-avian reptilian genomes provided by BGI and their collaborators (Table S9 in S1 Tables) were created using the Blast+ software package [75] and blasts searches (TBLASTN and BLASTp) were performed over these avian genomes to search for *Hh, LMBR1, RHEB* and *Trx/MLL2,3* coding sequences and relative locations annotated. All putative sequences identified were confirmed by TBLASTN and BLASTp over the GenBank [42] database.

Comparative *Dhh* gene synteny analysis across the reptilian group (birds and non-avian reptiles) was conducted by BLASTn of the GL343198.1 scaffold of the *Anolis carolinensis* anoCar2.0 assembly [105] over the *F. peregrinus* [106], *A. mississippiensis* [107] and *C. mydas* and the *Gallus gallus* WUGSC2.1 [108] assemblies. The results were confirmed by aligning the best-matched scaffolds/chromosomes with the lizard GL343198.1 scaffold using Mauve 2.3.1. [109], [110]. The localization of the *Dhh* gene and the conserved *LMBR1L–Dhh-RHEBL1-MLL2* cluster over the *Anolis carolinensis* genome was accessed from the GENOMICUS v64.01 browser [37], the complete genome assembly was downloaded from the UCSC database [111] and the subject scaffold extracted using UGENE 1.7.2 [112]. The complete *G. gallus* WUGSC2.1 assembly was downloaded also from the UCSC database [111] and local databases of the *F. peregrinus* and *G. gallus* genomes created using the Blast+ software package [75]. BLAST searches were performed using the Blast+ software package [75] and best hits chosen for Score, E-value and. Circular plots were created using Circos [113].

### 2. Sequence alignment and phylogenetic analyses

*Hh* coding sequences were retrieved from the GenBank [42] and Ensembl [38] databases and BLAST searches were used to recover non-annotated sequences from avian and other vertebrate genomes (Table S6 in S1 Tables). Local BLAST databases for 44 avian and two non-avian reptilian genomes provided by BGI-G10K and other groups (Table S9 in S1 Tables) were created using the Blast+ software package [75], and blasts searches (TBLASTN and BLASTp) were performed to search for *Hh* coding sequences. All putative sequences identified were confirmed by TBLASTN and BLASTp across the GenBank [42] database. We collected 120 *Hh* coding sequences and reduced the number to 50 by excluding sequences with less than 50% of the sites (relative to the *Homo sapiens* sequences) and equally represented each vertebrate class. A codon-based coding sequence alignment was constructed with the 50 sequences using MUSCLE 3.3 [76], manually adjusted using MEGA 5 [77] and viewed and edited in SEAVIEW [78]. It was previously reported that the alignment of *Hh* sequences produced indels on the C-terminal/3′ portion [10] and, as indels carry phylogenetic signal [79], filtering softwares were not applied. To assess the selective pressures acting on the three vertebrate *Hh* paralogs, four different alignments were produced: one for each paralog and a fourth with all sequences excluding outgroups. Nucleotide and amino acid conservation over *Hh* sequences was assessed using MEGA 5 [77].

For phylogenetic analyses, the substitution model that best fit our dataset (GTR+I+G) was selected using the Akaike Information Criterion (AIC) implemented in jModelTest [80], starting with 11 substitution schemes and using the fixed BIONJ-JC base tree for likelihood calculations. The dataset was checked for saturation bias in DAMBE [81], both by plotting the rate of transitions and transversions versus the genetic distance and by applying the Xia *et al.* test [82] to measure substitution saturation. This test compares half of the theoretical saturation index expected when assuming full saturation (I_SS.C_, critic value) with the observed saturation index (I_SS_). If I_SS_ is significantly lower than I_SS.C,_ the data has no evidence of saturation bias and can be further used for phylogenetic analysis. Although the saturation plot suggests a lower extent of substitution saturation, no statistically significant evidence of saturation was found in our dataset (S4 Fig. and Table S7 in S1 Tables). Therefore, the phylogeny was estimated using the Maximum Likelihood (ML) and Bayesian inference methods. The ML phylogenetic tree was constructed in PhyML 3.0 [83], with 1000 bootstrap replicates and the NNI branch search algorithm. Bayesian inference methods with Markov chain Monte Carlo (MCMC) sampling were preformed in MrBayes [84], [85], with 1,000,000 generations, a sample frequency of 100 and burn-in set to correspond to 25% of the sampled trees. For site tests of the *Hh* vertebrate paralogs, independent phylogenies for each gene were produced.

### 3. Adaptive selection detection

#### 3.1 Codon-level analysis

The four alignments produced and the ML/Bayesian trees were filtered with GBLOCKS 0.91 [86], [87] and used in the program codeml from the PAML v4.3 package [46] to evaluate adaptive evolution in the *Dhh, Ihh* and *Shh* coding sequences. To examine the ratio of non-synonymous substitutions per non-synonymous site (dN) to the number of synonymous substitutions per synonymous site (dS) (the dN/dS or ω ratio), the branch-specific and site-specific codon substitution models of maximum likelihood analysis were used.

For branch tests, four likelihood ratio-tests (LRT) were preformed to compare the log likelihood values of a two-ratio model, where the selected post-duplication branch has a different evolutionary rate relative to other branches (model = 2, NS sites = 0), against a one-ratio model, where all branches are supposed to evolve at a same rate (model = 0, NS sites = 0) [47]. The two-ratio (unconstrained two-ratio) model, if found to better fit the data, was tested against another null (constrained two-ratio) model where the ω_1_ value for the branch of interest was constrained to ω_1_<1 fixing ω_1_ = 1. The LRT between these two nested two-ratio models allows the detection of the prevalence of positive selection or relaxed selection constraints [47]. Hypothesis decision was performed assuming that LRT approximately follows the chi-square 2ΔlnL approximation (P<0.05), the double of the difference between the alternative and null model log likelihood [88]. LRT degrees of freedom were calculated as the difference of free parameters between the nested models. Individual two-ratio models were created using as foreground branch each one of the branches to test: the branch leading to the *Dhh* group, the branch leading to the *Ihh/Shh* group and the branches leading to the *Shh* and *Ihh* groups.

To detect signatures of adaptive evolution over the *Dhh*, *Ihh* and *Shh* codon sequences, three smaller phylogenetic trees were built for each group and each topology used for site analysis with PAML v4.03 [46]. Two LRTs were preformed to compare the log likelihood values of two nested models, a model that does not allow and a model that allows site positive selection [88]. First, the M0 (uniform selective pressure among sites; model = 0, NS sites = 0) and M3 (variable selective pressure among sites; model = 0, NS sites = 3) models were compared; and finally the M7 (beta distributed variable selective pressure; model = 0, NS sites = 7) and M8 (beta plus positive selection; model = 0, NS sites = 8) models. The identification of sites under positive selection was performed by Bayes Empirical Bayes (BEB) analysis [89].

The Single Likelihood Ancestor Counting (SLAC) and the Fixed Effects Likelihood (FEL) methods [49], implemented in the Datamonkey web server [50], [51], were used to detect signatures of purifying selection over the data. SLAC is a modified and improved derivative of the Suzuki-Gojobori counting approach that maps changes in the phylogeny to estimate selection on a site-by-site basis and it calculates the number of non-synonymous and synonymous substitutions that have occurred at each site using ML reconstructions of ancestral sequences [49], [50]. On the other hand, the FEL model estimates the ratio of non-synonymous to synonymous substitutions not assuming *a priori* distribution of rates across sites substitution on a site-by-site analysis [49].

Since the *Dhh* and *Shh* avian sequences, as well the turkey *Ihh* and lamprey *Hh* sequences, are incomplete (Table S9 in S1 Tables), these sequences were removed from the analysis, in order to improve the calculations and reduce the number of ambiguous sites.

#### 3.2 Amino acid-level analysis

To detect destabilizing selection signatures over *Dhh, Shh* and *Ihh* coding sequences, the three codon alignments and ML/Bayesian trees used for site-selection analysis where analyzed with the method implemented in TreeSAAP [56], finding which sites and significant physicochemical properties can be under positive and negative selection over the three analyzed lineages. TreeSAAP [56] compares the observed distribution of physicochemical changes inferred from the phylogenetic tree with an expected distribution based on the assumption of completely random amino acid replacement expected under the condition of selective neutrality. The evaluation of the magnitude of property change at non-synonymous residues and their location on a protein tridimensional structure may provide important information into the structural and functional consequences of the substitutions [52], [53].

Eight magnitude categories (1 to 8) represent one-step nucleotide changes in a codon and rank the corresponding variation on a property scale of the coded amino acid: categories 1 to 3 indicate stabilizing substitutions (small variations that tend to maintain the overall biochemistry of the protein) while categories 6 to 8 represent destabilizing substitutions (variations that result in radical structural and functional shifts in local regions of the protein). By accounting for the property changes across the data set, a set of relative frequency changes for each category is obtained, allowing the test of the null hypothesis under the assumption of neutral conditions: (1) positive selection is detected when the number of inferred amino acid replacements significantly exceeds the number expected by chance alone, resulting in positive Z-scores; (2) negative selection is detected when the expected number of amino acid replacement significantly exceeds those that are inferred, resulting in negative Z-scores [52], [53]. To detect both strong negative and positive selective pressures, only changes corresponding to categories 7 and 8 at the P≤0.05 (Z-score > |1.64|) and P≤0.001 (Z-score > |3.09|) levels were considered, due to the strong purifying signatures over our data. A total of 31 amino acid properties [53] were evaluated for each paralog and, to verify which specific regions were affected by negative and positive destabilizing selection, we performed a sliding window analysis using the properties which were significant for the signal. Sliding windows of 10 amino acid length with a sliding step of one codon were selected to show the best signal-to-noise ratio and to identify regions in the vertebrate *Hh* proteins that differ significantly from a nearly neutral model [90]. In addition, we identified the total number of changes per site assuming it as the sum of those occurring in each branch of the phylogeny [54].

### 4. Functional divergence analysis

The detection of functional divergence was carried out with DIVERGE 2.0 [60], using the Gu2001 method [58] for Type I functional divergence and the Gu *et al.* method [59] for Type II functional divergence. Type I functional divergence represents amino acid residues that are universally conserved through one subfamily but highly variable in another, implying that these residues have experienced altered functional constraints after duplication [58]. On the other hand, Type II functional divergence represents amino acid configurations that are much conserved in each subfamily but whose biochemical properties are very different, implying that these residues may be responsible for functional specification [59].

The coefficient of Type I and Type II functional divergence (θ_I_ and θ_II_) between each pair of *Hh* paralogs were estimated. A parameter significantly greater than zero means that either altered selective constraints or a radical shift of amino acid physicochemical properties after gene duplication were likely to have occurred. LRT calculations for the null hypothesis (i.e., the absence of functional divergence) were performed to assess the significance of the parameter. In order to detect which residues are more likely to be responsible for functional divergence, the posterior probability [P(S_1_|X)] for the functional divergence for each position in the alignment was calculated. The cut-off value for the posterior probability was first set to P(S_1_|X) >0.5, which corresponds to a posterior odd ratio R(S_1_|S_0_) = P(S_1_|X)/P(S_0_|X) >1 and to a meaningful evidence [91]. A more stringent cutoff was selected based on the Harold Jeffreys scale for interpretation of R(S_1_|S_0_), selecting P(S_1_|X) ≥0.91 as it corresponds to R(S_1_|S_0_) ≥10 (strong evidence) [92].

### 5. Protein structural modeling and manipulation

Only the tridimensional structures of the two separated Hedgehog domains are currently available on the Protein Data Bank (PDB) [61], [93]: the human and murine ShhN, IhhN and DhhN regions and the *Drosophila melanogaster* HhN and HhC domains. Thus, we used the 3HO5 (Human ShhN), 2WFR (Human DhhN) and the 3K7G (Human IhhN) PDB files in order to represent the Hedge domain of the human Hh proteins and modeled the tridimensional structure of the human ShhC, IhhC and DhhC domains using I-TASSER [94]. I-TASSER is a platform for protein tridimensional structure and function prediction implemented on the I-TASSER server [95] that combines *ab initio* and comparative modeling approaches to generate a high quality tridimensional model and has been ranked as the best method for automated protein structure prediction in the last CASP experiments [96]–[101].

The I-TASSER platform measures the quality of the generated model using two different scoring functions: (1) the C-score is a confidence score for estimating the quality of the predicted models and it is calculated based on the significance of threading template alignments and the convergence parameters of the structure assembly simulations [102]; (2) the TM-score is a scale for measuring the structural similarity between two structures and is used to measure the accuracy of structure modeling when the native structure is known in order to test if the result topology is not random [103]. As in these cases the native structure is not known, the TM-score is calculated based on the C-score [102]. To accurately infer the correct topology, the model should have a C-score above −1.5, varying from [-5;2], and TM-score above 0.5 [102], [103] (Table S8 in S1 Tables). Visualization and manipulation of the generated models, as well as root-mean-square deviation (RMSD) deviation values determination, were assessed with PyMol [104].

## Conclusions

In addition to the characterization of the three *Hh* vertebrate paralogs in newly sequenced avian and non-avian reptilian species, this work provided new insights into the evolutionary history of the *Hh* gene family after two independent WGD events. We showed that in contrast with invertebrates, vertebrates experienced different evolutionary fates and evolved under different selective constraints after duplication. The structural regions around the ion binding site that were identified to be under positive selection at the signalling domain may provide new insights into the functional roles and expression patterns of these paralogs in vertebrate species and lead to the discovery of new mutational hotspots. It will also be interesting to reanalyze our results with complete, instead of processed, vertebrate Hh-protein tridimensional structures. We could then assess if residues identified under positive and negative selection that are on the protein surface and that are separate from the active sites (Hedge ion binding site and Hog catalytic site) are responsible for interactions formed between both domains before processing, thus enhancing the reaction, or if they are only involved in helping maintain an intact domain structure.

## Supporting Information

S1 Fig **Amino acid configurations of the sites with a type I functional divergence posterior probability P(S_1_|X) 0.91 for each pair of vertebrate Hh paralog proteins.**
(TIF)

S2 Fig **Tridimensional arrangement of negatively and positively regions over the Hog domain of vertebrate Hedgehog proteins.** Proteins (ShhN: PDB 3HO5, DhhN: PDB 2WFR; IhhN: PDB 3K7G) represented in grey cartoon with transparent surface. Negatively selected sites (green) identified with FEL, positively selected regions for the amino acid isoelectric point property (orange) and positively selected sites (red) identified with TreeSAAP are shown for each paralog domain. A dashed circle denotes the position of the calcium/zinc binding-site.
(TIF)

S3 Fig **Tridimensional arrangement of negatively and positively regions over the Hedge domain of vertebrate Hedgehog proteins.** Proteins (modelled by homology with the human sequence for each paralog, using *Drosophila melanogaster* Hh protein as a template on I-TASSER) represented in grey cartoon with transparent surface. Negatively selected sites (green) identified with FEL, positively selected regions for the amino acid isoelectric point property (orange) and positively selected sites (red) identified with TreeSAAP are shown for each paralog domain. Arrows marks those residues surrounding the 324 codon alignment position. A dashed circle denotes the position of the catalytic site.
(TIF)

S4 Fig **Nucleotide saturation plot for coding sequences of vertebrate** ***Hh*** **paralogs.** Representation of transitions (s) and transversions (v) at all three codon positions versus the genetic distance retrieved by the GTR nucleotide substitutions model.
(TIF)

S1 Tables **Table S1.** Positively and negatively selected residues detected by SLAC and FEL. The percentage and the numbering of the negatively selected residues is in agreement with the Homo sapiens sequences and residues associated to a disease or to a functional processe, as annotated on the GenBank and UniProt databases, are highlighted. **Table S2.** Sites detected by SLAC and FEL with dN/dS values above 1 but not statistically positively selected. **Table S3.** Sites under strong positive selection (p<0.001) on the three vertebrate Hh paralogs, according to TreeSAAP. Site numbering refers to the Homo sapiens sequences and an asterisk marks the sites which were detected as under negative selection with SLAC ad FEL. **Table S4.** Estimates of the coefficient of fucntional divergence type I (θI) calculated with DIVERGE 2.0 for each pair of vertebrate Hh paralog proteins. **Table S5.** Amino acid residues with a type I functional divergence posterior probability P(S1|X)≥0.91 for each pair of vertebrate Hh paralog proteins, calculated with DIVERGE 2.0. The site position in each alignment is listed, as well the correspondent position on the human protein sequence. Homo sapiens First refers to the first member of the pair, and Homo sapiens Second to the second member of the pair. An asterisk (*) marks negatively selected residues presented on Table S1. **Table S6.** List of Hh sequences collected from currently available genomes. **Table S7.** Test of substitution saturation by Xia et al. using DAMBE. Analysis performed on fully resolved sites only, testing whether the observed Iss is significantly lower than Iss.c. IssSym is Iss.c. assuming a symmetrical topology; IssAsym is Iss.c. assuming an asymetrical topology. **Table S8.** Quality scores for modelled ShhC, IhhC and DhhC protein domains, determined using I-TASSER. **Table S9.** List of studied non-avian and avian reptile species included on the BGI Birs Phylogenomic Project. The taxonomic classification of each species was retrieved from the Integrated Taxonomic Information System on-line database (ITIS) (http://www.itis.gov/).
(XLSX)

S1 Material **Supplementary material.**
(DOCX)
