# Supplementary figures and images for: Evolutionary Genomics and Adaptive Evolution of the Hedgehog Gene Family (Shh, Ihh and Dhh) in Vertebrates
Source: PLoS One. 2014 Dec 30;9(12):e74132. doi: 10.1371/journal.pone.0074132 (PMC4280113; doi:10.1371/journal.pone.0074132)

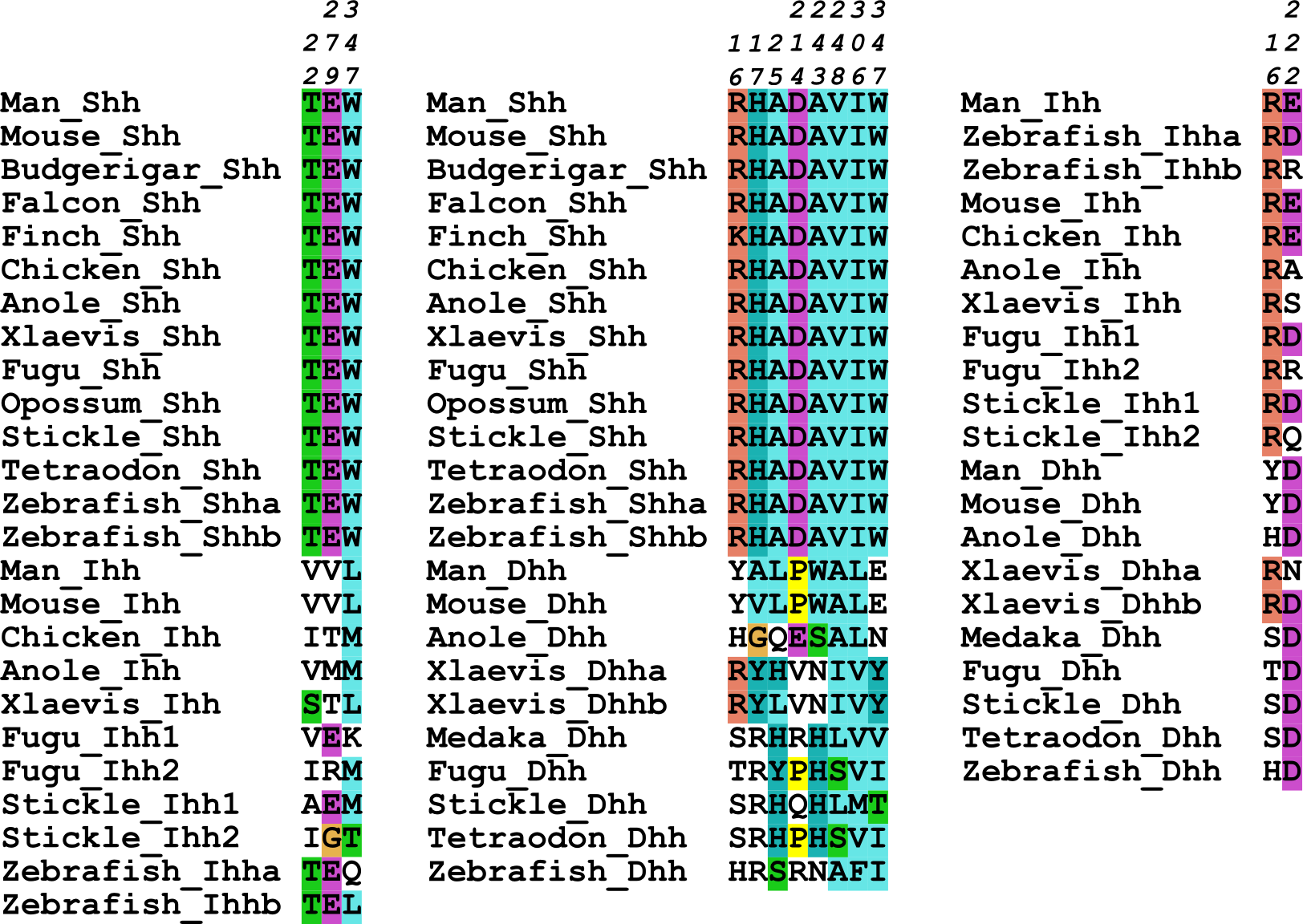

Supplement: S1 Fig — Amino acid configurations of the sites with a type I functional divergence posterior probability P(S1|X) 0.91 for each pair of vertebrate Hh paralog proteins. (TIF) [file pone.0074132.s001.tif]

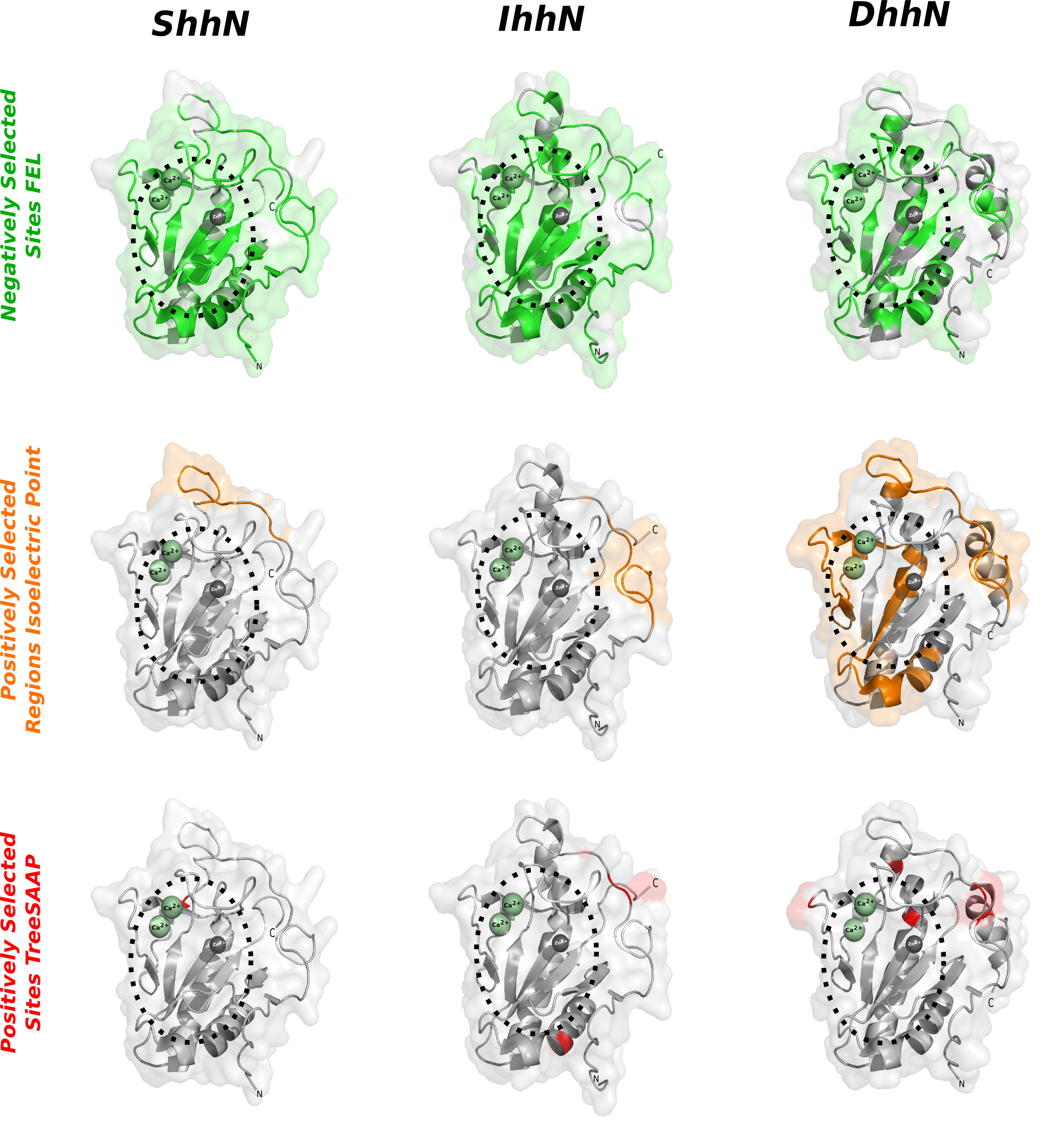

Supplement: S2 Fig — Tridimensional arrangement of negatively and positively regions over the Hog domain of vertebrate Hedgehog proteins. Proteins (ShhN: PDB 3HO5, DhhN: PDB 2WFR; IhhN: PDB 3K7G) represented in grey cartoon with transparent surface. Negatively selected sites (green) identified with FEL, positively selected regions for the amino acid isoelectric point property (orange) and positively selected sites (red) identified with TreeSAAP are shown for each paralog domain. A dashed circle denotes the position of the calcium/zinc binding-site. (TIF) [file pone.0074132.s002.tif]

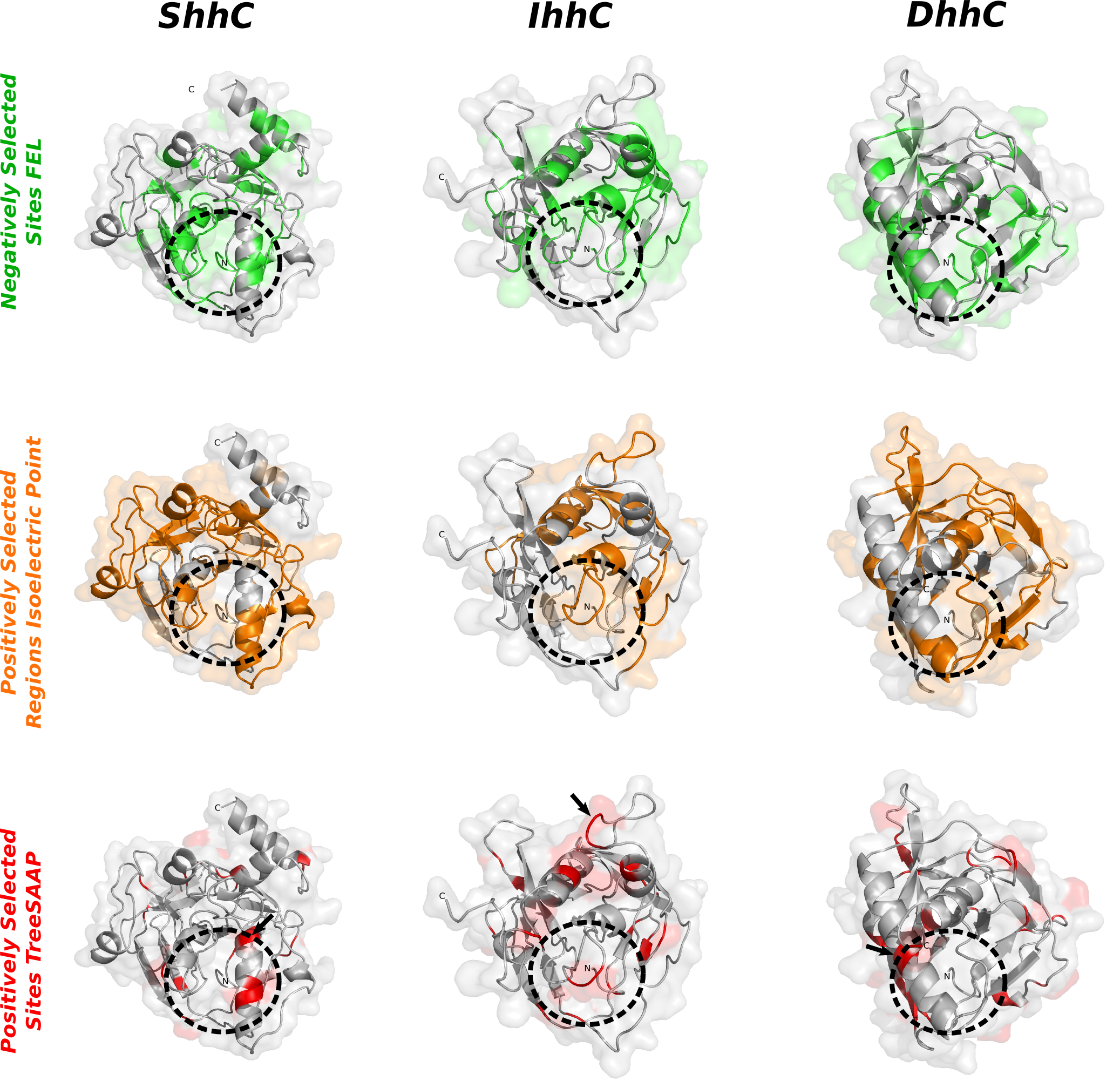

Supplement: S3 Fig — Tridimensional arrangement of negatively and positively regions over the Hedge domain of vertebrate Hedgehog proteins. Proteins (modelled by homology with the human sequence for each paralog, using Drosophila melanogaster Hh protein as a template on I-TASSER) represented in grey cartoon with transparent surface. Negatively selected sites (green) identified with FEL, positively selected regions for the amino acid isoelectric point property (orange) and positively selected sites (red) identified with TreeSAAP are shown for each paralog domain. Arrows marks those residues surrounding the 324 codon alignment position. A dashed circle denotes the position of the catalytic site. (TIF) [file pone.0074132.s003.tif]

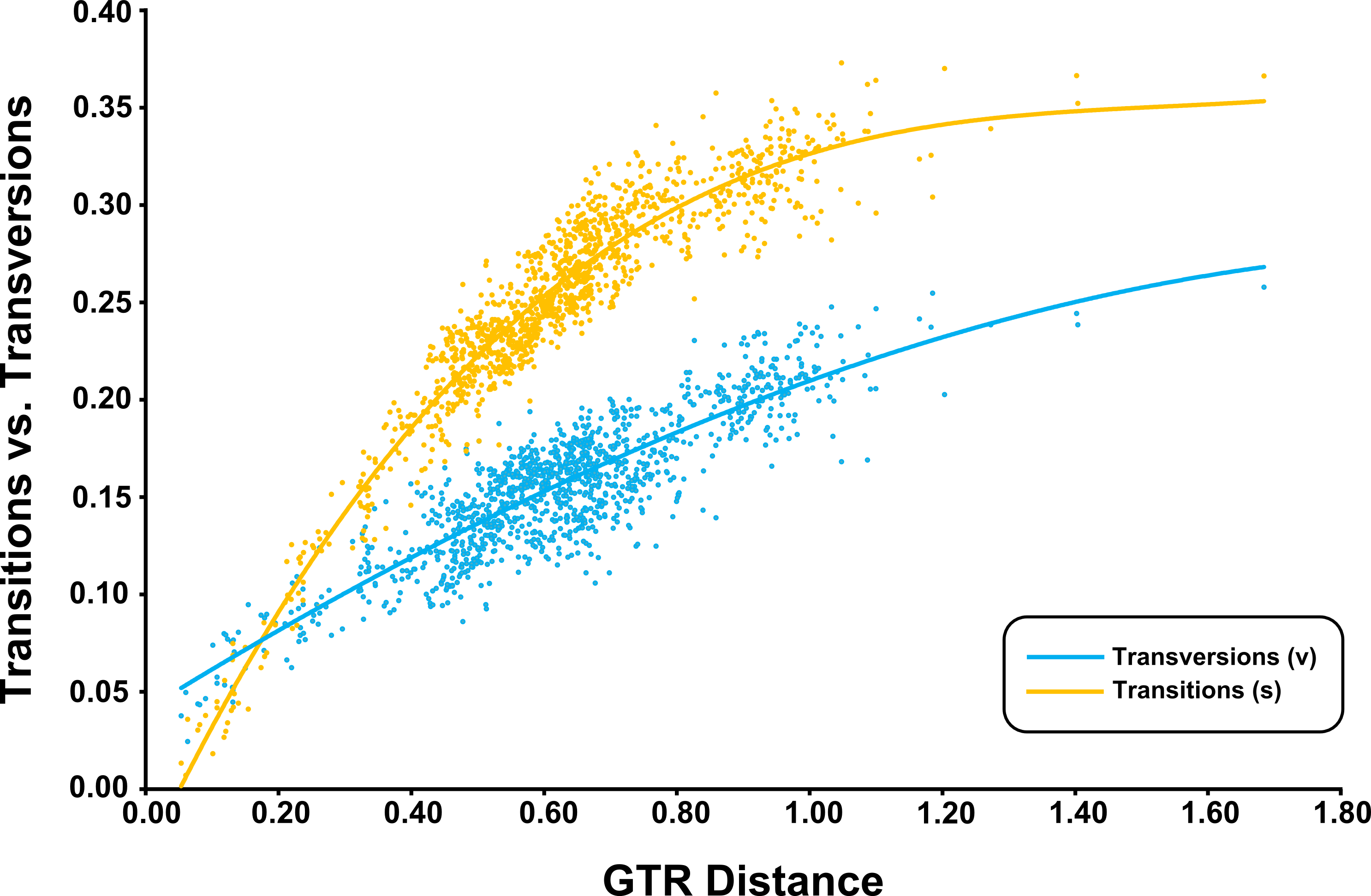

Supplement: S4 Fig — Nucleotide saturation plot for coding sequences of vertebrate Hh paralogs. Representation of transitions (s) and transversions (v) at all three codon positions versus the genetic distance retrieved by the GTR nucleotide substitutions model. (TIF) [file pone.0074132.s004.tif]
