# Supplementary material for: Evolutionary Genomics and Adaptive Evolution of the Hedgehog Gene Family (Shh, Ihh and Dhh) in Vertebrates
Source: PLoS One. 2014 Dec 30;9(12):e74132. doi: 10.1371/journal.pone.0074132 (PMC4280113; doi:10.1371/journal.pone.0074132)
Supplement: S1 Material — Supplementary material. (DOCX) [file pone.0074132.s006.docx]

**Supplementary Material 1**

***Evolution at the Genomic Level***

In order to address why a *Dhh* paralog is missing on the currently and publicaly available avian genomic data, we determined the synteny of the *Dhh* gene in the genomes of several species that represent major groups of vertebrates using the GENOMICUS v64.01 database browser [[37](#_ENREF_37)] and 48 genomes from avian and non-avian reptilian species provided by the BGI-G10K avian Phylogenomics Project (paper in preparation), and compared it with the synteny of the other two vertebrate members of the *Hh* gene family (Fig. 2). We observed that the *Dhh* gene formed a conserved linkage group with the *LMBR1L, RHEBL1* and *MLL2* genes in all the tetrapods in the database. Teleost fishes had a similar group composed of the *LMBR1L*, *Dhh* and *MLL2* genes, where the *Dhh* and *MLL2* genes are adjacent to each other and the *RHEBL1* gene is more-separated.

Using solely data from the databases, only *LMBR1L* and a *MLL2* gene were found in the genome of the Neoaves (*Taeniopygia gutatta*). These were located on the same partially assembled chromosome (Un_random), separated by a large gap of genes. In Galloanserae (*Gallus gallus* and *Meleagris gallopavo*) only *MLL2* was annotated (Fig. 2) but BLAST (TBLASTN and BLASTp) searches over the 45 genomes from avian and non-avian reptilian species showed evidence of all the genes that comprise this cluster in this tetrapod lineage (Table S9). The best results were obtained from the Neoavian species *Falco peregrinus* (peregrine falcon), *Melopsittacus undulatus* (budgerigar) and *Haliaeetus leucocephalus* (bald eagle) genomes and two non-avian reptiles, *Alligator mississippiensis* (american alligator) and *Chelonia mydas* (green turtle) (Table S9). In *F. peregrinus* and *C. mydas* the conserved tetrapod *LMBR1L-Dhh-RHEBL1-MLL2* cluster was found on scaffolds 373.1 and 429, respectively (Fig. 3A). In *M. ondulatus,* *H. leucocephalus* and *A. mississippiensis,* all four genes were identified, but they were dispersed on small scaffolds.

Comparing the synteny of the *Dhh* gene in the lizard (*A. carolinensis*) and turtle (*C. mydas*)*,* with the birds *F. peregrinus* and *G. gallus* by BLAST searches, we found four main turtle scaffolds and six falcon scaffolds that had high similarity with specific regions of the lizard GL343198.1 scaffold (Fig. 3B and C), while in the chicken genome we found similarity with regions of two macrochromosomes, a linkage group and the Un_random chromosome (Fig. 3C). Although the correspondence among the genomes was clear for the portions of the lizard GL343198.1 scaffold that are outside the region of *Dhh* synteny, it was more difficult to discern clear correspondence within the *Dhh* and syntenic regions (Fig. 3B, C and D). This may be because upstream of the *LMBR1L* gene on the lizard scaffold there are three genes that are members of the Tubulin-α family [[37](#_ENREF_37),[38](#_ENREF_38)], a highly conserved and gene-rich family coding for an important structural family of proteins [[39](#_ENREF_39),[40](#_ENREF_40)]. As expected, we found matches beyond this region on a turtle scaffold (429) and falcon scaffold (373.1), without dispersed matches, but with the chicken assembly the best matches were with the partially assembled Un_random chromosome, with many highly dispersed hits (Fig. 3B, C and D).

The regions of the lizard GL343198.1 scaffold just downstream and upstream of the best matches on the *C. mydas* 429 scaffold (Fig. 3B), also match specific regions of scaffold 438 of the *C. mydas* assembly. This result could have resulted from a rearrangement over this region in the two reptilian species. Similarly, in *F. peregrinus* the 350.1 scaffold was identical to a region slightly downstream of the *Dhh* syntenic region (Fig. 3C), while in the *G. gallus* assembly this region had high sequence similarity with part of the E22C19w28_E50C23 linkage group (Fig. 3D). Using the 429 and 438 *C. mydas* scaffolds, and the 350.1 and 373.1 *F. peregrinus* scaffolds as queries in a BLASTn of a local database of the *G. gallus* genome, we found that a large region from the 5’ end of the 429 turtle scaffold has high identity with the 5’ end of the chicken E22C19w28_E50C23 linkage group, while the 3’ end of the 429 scaffold matched with several regions of the chicken Un_random chromosome (Fig. 3E). Inversely, a large part of the 438 turtle scaffold has high similarity with random regions of the chicken Un_random chromosome and a few sequences from the E22C19w28_E50C23 linkage group (Fig. 3E).

In *F. peregrinus,* the 373.1 scaffold had high similarity with several random regions of the *G. gallus* Un_random chromosome, which also matched portions of the turtle scaffolds 429 and 438 (Fig. 3E and F). However, its 5’ end had high similarity with the 3’ end of the *G. gallus* E22C19w28_E50C23 linkage group. Similarly, the *F. peregrinus* 350.1 scaffold had regions with high similarity with random positions on the *G. gallus* Un_random chromosome, as well as with two regions of the *G. gallus* E22C19w28_E50C23 linkage group, one located near the 373.1 scaffold hit. This suggests that the *F. peregrinus* 373.1 and 350.1 matches may assemble with each other. However, when the assembly and alignment of both scaffolds was performed, it was not possible to build a sequence as no contig was found. On the other hand, when we looked at the region of the 373.1 scaffold where the *Dhh* and syntenic genes were found on the falcon genome (Fig. 3G), we found matches in the *G. gallus* genome only for the falcon *MLL2* gene, which is highly dispersed on the chicken Un_random chromosome.
